# Roles and interplay of reinforcement-based and error-based processes during reaching and gait in neurotypical adults and individuals with Parkinson’s disease

**DOI:** 10.1371/journal.pcbi.1012474

**Published:** 2024-10-14

**Authors:** Adam M. Roth, John H. Buggeln, Joanna E. Hoh, Jonathan M. Wood, Seth R. Sullivan, Truc T. Ngo, Jan A. Calalo, Rakshith Lokesh, Susanne M. Morton, Stephen Grill, John J. Jeka, Michael J. Carter, Joshua G. A. Cashaback

**Affiliations:** 1 Department of Mechanical Engineering, University of Delaware, Newark, Delaware, United States of America; 2 Department of Biomedical Engineering, University of Delaware, Newark, Delaware, United States of America; 3 Kinesiology and Applied Physiology, University of Delaware, Newark, Delaware, United States of America; 4 Biomechanics and Movement Science Program, University of Delaware, Newark, Delaware, United States of America; 5 Department of Physical Therapy, University of Delaware, Newark, Delaware, United States of America; 6 Interdisciplinary Neuroscience Graduate Program, University of Delaware, Newark, Delaware, United States of America; 7 Johns Hopkins Regional Physicians, Johns Hopkins University, Baltimore, Maryland, United States of America; 8 Department of Kinesiology, McMaster University, Hamilton, Ontario, Canada; Pennsylvania State University Main Campus: The Pennsylvania State University - University Park Campus, UNITED STATES OF AMERICA

## Abstract

From a game of darts to neurorehabilitation, the ability to explore and fine tune our movements is critical for success. Past work has shown that exploratory motor behaviour in response to reinforcement (reward) feedback is closely linked with the basal ganglia, while movement corrections in response to error feedback is commonly attributed to the cerebellum. While our past work has shown these processes are dissociable during adaptation, it is unknown how they uniquely impact exploratory behaviour. Moreover, converging neuroanatomical evidence shows direct and indirect connections between the basal ganglia and cerebellum, suggesting that there is an interaction between reinforcement-based and error-based neural processes. Here we examine the unique roles and interaction between reinforcement-based and error-based processes on sensorimotor exploration in a neurotypical population. We also recruited individuals with Parkinson’s disease to gain mechanistic insight into the role of the basal ganglia and associated reinforcement pathways in sensorimotor exploration. Across three reaching experiments, participants were given either reinforcement feedback, error feedback, or simultaneously both reinforcement & error feedback during a sensorimotor task that encouraged exploration. Our reaching results, a re-analysis of a previous gait experiment, and our model suggests that in isolation, reinforcement-based and error-based processes respectively boost and suppress exploration. When acting in concert, we found that reinforcement-based and error-based processes interact by mutually opposing one another. Finally, we found that those with Parkinson’s disease had decreased exploration when receiving reinforcement feedback, supporting the notion that compromised reinforcement-based processes reduces the ability to explore new motor actions. Understanding the unique and interacting roles of reinforcement-based and error-based processes may help to inform neurorehabilitation paradigms where it is important to discover new and successful motor actions.

## Author summary

Reinforcement-based and error-based processes play a pivotal role in regulating our movements. Converging neuroanatomical evidence show interconnected reinforcement-based and error-based neural circuits. Yet is unclear how reinforcement-based and error-based processes interact to influence sensorimotor behavior. In our past work we showed that reinforcement-based and error-based processes are dissociable. Building on this work, here we show that these process can also interact to influence trial-by-trial sensorimotor behaviour.

## Introduction

From a toddler taking their first steps to an adult relearning a sensorimotor skill following a neurological disease, exploration is critical to discovering successful motor actions [1, 2]. We often receive reinforcement feedback (knowledge that an action was successful) or error feedback (the direction and magnitude of a movement error) for our actions. Reinforcement feedback can promote exploration by encouraging new actions to find success [3–7]. Conversely, error feedback is used to make corrective motor actions that improve accuracy [8–12], which may impact exploratory behaviour [8]. Currently it is unclear how reinforcement-based and error-based processes uniquely contribute and or interact to influence sensorimotor exploration. Understanding the processes that underpin sensorimotor exploration may lead to more informed neurorehabilitation paradigms that aim to discover new and successful functional motor skills [1, 2, 13, 14].

Exploratory behaviour in songbirds has been linked to positive reinforcement (i.e., reward) and the basal ganglia [15–17]. During human reaching, we recently showed that reinforcement-based processes actively regulate sensorimotor exploration along task-redundant dimensions [3]. In this paradigm, participants were told to reach and stop within a long rectangular target. The major axis of the target was task-redundant and encouraged exploration. Conversely, the minor axis of the target was task-relevant. Participants received binary positive reinforcement feedback when they successfully stopped within the target. Investigating exploration along task-redundant dimensions can be useful to isolate exploratory mechanisms. We quantified exploratory random walk behaviour along the task-redundant dimension using lag-1 autocorrelation, which is ubiquitously seen in both reaching [3, 8, 18, 19] and gait [20, 21] behaviour. Another metric that has been used to quantify exploration is the variability of the trial-by-trial change in reach position [3, 4, 6, 18, 22–24]. Work by Pekny and colleagues (2015) showed that this trial-by-trial variability increases following an unsuccessful movement. Further, Pekny and colleagues (2015) showed that this modulation of trial-by-trial variability is reduced in individuals with Parkinson’s disease. Parkinson’s disease is caused by neuronal death in the basal ganglia that impacts reinforcement (reward) processes and associated pathways [25, 26]. Thus, using a clinical model of Parkinson’s disease is a powerful way to gain mechanistic insight into the role of reinforcement-based neural processes on exploratory motor behaviour.

Just as exploration in response to reinforcement-based feedback is linked to the basal ganglia, movement corrections in response to error-based feedback are predominantly attributed to the cerebellum [27–31]. A greater magnitude of movement variability is commonly observed along task-redundant compared to task-relevant dimensions [8, 32–37]. Elegant empirical and theoretical work by van Beers and colleagues (2013) investigated exploratory random walk behaviour along task-redundant dimensions compared to task-relevant dimensions. In their task, participants were told to reach and stop close to a thin line target. In this paradigm, the major axis of the target was task-redundant, while the minor axis of the target was task-relevant. Participants received error feedback each trial in the form of a visual cursor at their final hand position. Van Beers and colleagues observed greater lag-1 autocorrelation along the task-redundant dimensions compared to the task-relevant dimension, indicating heightened exploratory random walk behaviour. The authors attributed this to an accumulation of planned noise during the planning stages of movement [38, 39] and a lack of error correction towards the center of the target. Thus, it is possible that exploratory behaviour can arise passively through a lack of error-based processes correcting movement aim [8] and/or reinforcement-based processes actively updating movement aim towards recently successful actions [3].

There is converging neuroanatomical evidence suggesting that reinforcement-based and error-based processes interact. The basal ganglia and cerebellum share direct connections with one another [40–43], as well as interconnections to the same motor planning circuitries such as the dorsal premotor [44] and prefrontal [45] cortices. Further, signatures of reward have been found in the cerebellum [46, 47], and the cerebellum has been shown to directly modulate dopaminergic activity [43]. While our past work has shown that these reinforcement-based and error-based processes are dissociable during adaptation [48], it is unknown whether they interact to influence sensorimotor behaviour.

Here we hypothesize that reinforcement-based processes boost exploratory behaviour by updating movement aim towards a successful action, while error-based processes suppress exploratory behaviour by correcting movement aim. When acting in concert, we hypothesize these reinforcement-based and error-based processes will mutually oppose one another to impact sensorimotor exploration. For all three experiments, we made *a priori* predictions with a general model that incorporated both reinforcement-based and error-based mechanisms. In **Experiment 1**, we investigated the individual roles of reinforcement-based and error-based processes on sensorimotor exploration. We predicted that participants would display greater exploratory random walk behaviour when receiving reinforcement feedback compared to error feedback. Our findings in reaching during **Experiment 1** generalized to walking, which we found by re-analyzing a recent gait study [49]. In **Experiment 2**, we replicated the results of **Experiment 1** while also investigating how reinforcement-based and error-based processes act in concert to influence sensorimotor exploration. When given simultaneous reinforcement & error feedback, we expected that exploratory random walk behaviour would be greater than when given isolated error feedback but less than when given isolated reinforcement feedback. In **Experiment 3**, we gained mechanistic insight into the role of reinforcement-based neural processes in sensorimotor exploration by recruiting participants with Parkinson’s disease and age-matched control participants. We predicted participants with Parkinson’s disease would display less exploratory random walk behaviour when given reinforcement feedback compared to age-matched control participants. We then found the best-fit model from nine different plausible models, each testing different error correction mechanisms while including reinforcement-based terms from our previous work [3]. Taken together, our empirical results and modelling work suggest that reinforcement feedback boosts exploration and is causally linked to the basal ganglia and associated reinforcement (reward) processes. Additionally, our results suggest that error feedback suppresses exploration, while the interaction of reinforcement feedback & error feedback interact to impact sensorimotor exploration.

## Results

### Experimental design

**Fig 1:**
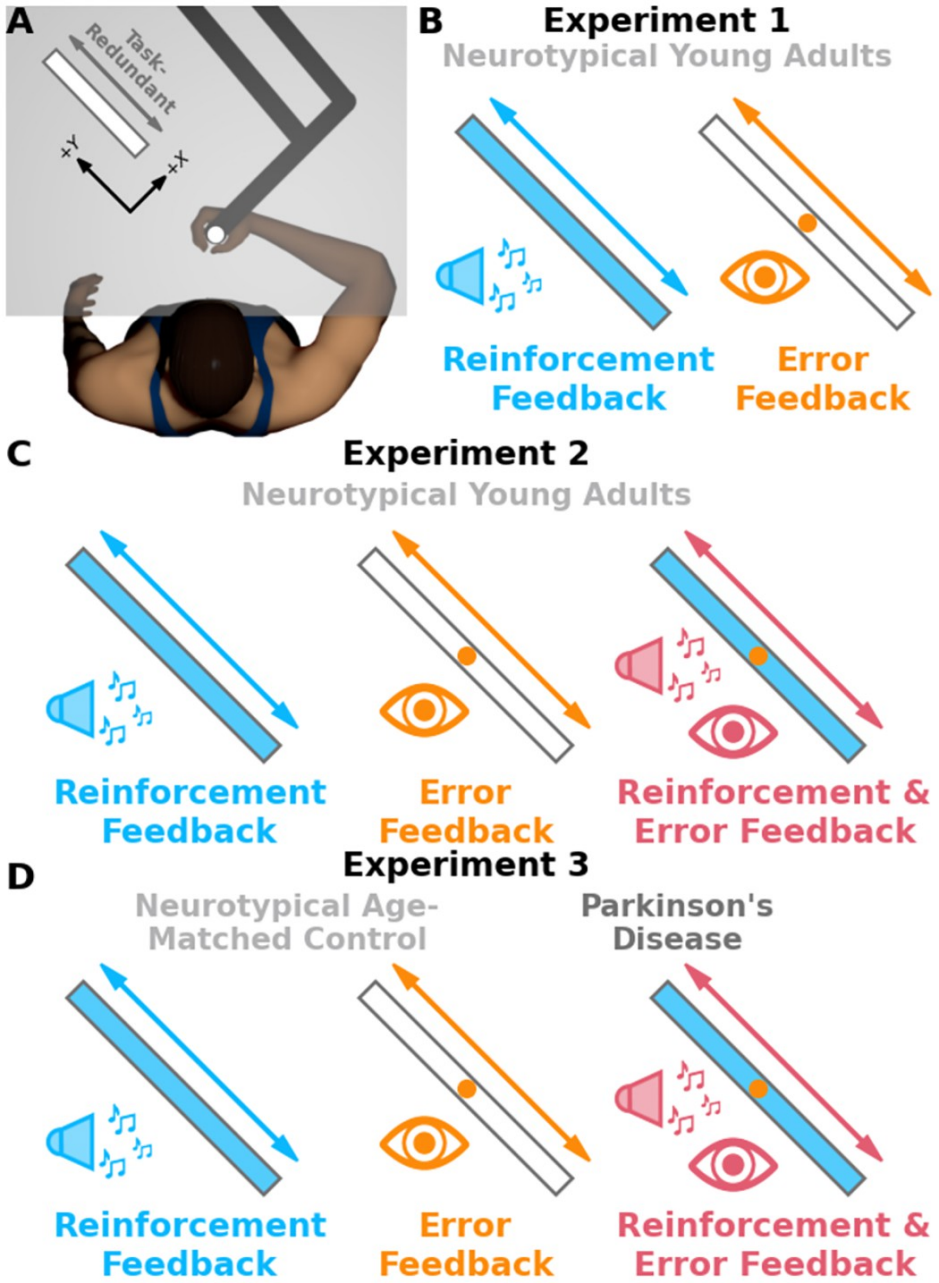
Experimental design. In all experiments, participants grasped the handle of a robotic manipulandum and made reaching movements in the horizontal plane. An LCD display projected images (start position, targets) onto a semi-silvered mirror that occluded vision of the hand and upper arm. **A)** Participants were told to reach from a start position (white circle) and stop within a target (white rectangle) that promoted exploration along the task-redundant dimension. **B)** In **Experiment 1**, we investigated how reinforcement-based and error-based processes differentially impact sensorimotor exploration. Participants experienced two conditions. In one condition, participants received isolated reinforcement feedback (sound icon, blue) when their hand successfully stopped within the target. With reinforcement feedback, participants heard a pleasant noise, the target turned blue, and they received a small monetary reward. In the other condition, participants received isolated error feedback (eye icon, orange). With error feedback, participants saw a cursor appear at their final hand position. **C)** In **Experiment 2**, we sought to replicate the results of **Experiment 1** while investigating how reinforcement-based and error-based processes interact during sensorimotor exploration. **Experiment 2** had three conditions: reinforcement feedback (blue), error feedback (orange), and simultaneously both reinforcement & error feedback (pink). **D)** In **Experiment 3**, our goal was to gain mechanistic insight into the role of the basal ganglia and associated reinforcement pathways on sensorimotor exploration. Those with Parkinson’s disease have impaired reinforcement-based neural circuitry from neuronal death in the basal ganglia. Thus, we recruited neurotypical age-matched control (light grey) participants and those with Parkinson’s disease (dark grey). Participants in **Experiment 3** also performed three conditions: reinforcement feedback (blue), error feedback (orange), and reinforcement & error feedback (pink).

For all experiments, participants made targeted reaching movements in the horizontal plane while holding the handle of a robotic manipulandum (Fig 1A, KINARM, BKIN Technologies, Ontario, Canada). Images of the start position, target, and cursor were projected onto a silvered mirror that occluded vision of the hand and arm. Participants began each trial by moving the handle of a robotic manipulandum to a start position. Participants were instructed to reach and stop within a virtually displayed target, without vision of their hand. We recorded the final hand position for each reach after participants stopped their hand within or near the virtually displayed target. In **Experiment 1** (Fig 1B), we investigated how reinforcement-based and error-based processes differentially influence exploration along task-redundant dimensions. We predicted that participants would display greater exploration when receiving reinforcement feedback compared to error feedback. Thirty-six young, neurotypical participants performed 50 baseline, 200 experimental trials, 50 washout trials, and another 200 experimental trials.

During baseline and washout blocks, participants attempted to reach and stop within a small white circle. Participants saw a small yellow cursor after stopping their hand within or near the small white circle for the first 40 trials of baseline and washout. Participants received no feedback for the last 10 trials of baseline and washout. Removing feedback for the last 10 during baseline allowed us to estimate trial-by-trial movement variability without the influence of corrective actions.

During experimental trials, participants attempted to reach and stop within a large rectangular target (Fig 1A). The major axis of the target was aligned with the movement extent. In this task, the major axis represents the task-redundant dimension and encourages exploratory behaviour. The minor axis of the target was scaled based on participant movement variability during the last 10 trials of baseline [3, 18]. Scaling target width based on individual movement variability ensured similar task difficulty across participants. Participants experience two conditions: isolated reinforcement feedback and isolated error feedback. With reinforcement feedback, each time participants successfully stopped within the target they would receive a small monetary reward, hear a pleasant sound, and see the target expand and change colour. Participants received no feedback if their final hand position was outside the target during the reinforcement feedback condition. With error feedback, a small yellow cursor would appear at the participant’s final hand position. Condition order was counterbalanced.

In **Experiment 2** (Fig 1C), we sought to replicate the results of **Experiment 1** while also investigating the interplay between reinforcement-based and error-based processes. As in **Experiment 1**, we predicted that participants would display greater explorative behaviour when receiving reinforcement feedback compared to error feedback. We also predicted that participant explorative behaviour under reinforcement & error feedback would be greater than isolated error feedback, but less than isolated reinforcement feedback. In **Experiment 2**, thirty-six young neurotypical participants performed 50 baseline trials, 200 experimental trials, 50 washout trials, 200 experimental trials, 50 washout trials, and another 200 experimental trials. Baseline and washout trials were identical to **Experiment 1**. Participants experienced three conditions: isolated reinforcement feedback, isolated error feedback, and simultaneous reinforcement & error feedback. Condition order was counterbalanced.

In **Experiment 3** (Fig 1D), we sought to gain mechanistic insight into the role of the basal ganglia in exploratory behaviour along task-redundant dimensions. We recruited participants with Parkinson’s disease as a population with a known compromise to the basal ganglia, as well as neurotypical age-matched control participants. Participants with Parkinson’s disease (N = 10, age: 68.4 ± 8.4 years) and neurotypical age-matched control participants (N = 12, age: 69.7 ± 6.9 years) performed three conditions: isolated reinforcement feedback, isolated error feedback, and simultaneous reinforcement & error feedback. Participants performed 50 baseline trials, 100 experimental trials, 50 washout trials, 100 experimental trials, 50 washout trials, and another 100 experimental trials. Baseline and washout trials were identical to **Experiment 1**. Baseline movement variability was not significantly different between the Parkinson’s disease group and age-matched control group along either the minor (p = 0.82) or major (p = 0.51) target axes. Trial count was reduced from **Experiment 2** to minimize the potential influence effects of fatigue for the older population. Condition order was randomized. We predicted that participants with Parkinson’s disease would display less explorative behaviour when given reinforcement feedback compared to neurotypical age-matched controls.

### *A Priori* model predictions

Previous work by van Beers and colleagues (2013) investigated exploratory random walk behaviour along task-redundant dimensions. Random walk behaviour is a statistical characteristic of time-series data that captures the temporal relationship between data points. In their task, participants received error feedback while reaching to a long thin line target. The major target axis corresponded to the task-redundant dimension while the minor axis corresponded to the task-relevant dimension. Participants displayed greater random walk behaviour (lag-1 autocorrelation) along the task-redundant dimension compared to the task-relevant dimension. The authors attributed this finding to an accumulation of planned noise during the planning stages of movement [38, 39] and a lack of error corrections along the task-redundant dimension. While the amount of planned movement variability resulting from planned noise may be small on a single trial, in their model planned movement variability is added to the intended movement aim on each trial. Thus, over many trials, planned movement variability accumulates in the intended movement aim. The accumulation of this small amount of movement variability over many trials, without any form of error-based correction, could result in a large drift away from the original intended movement aim. However, it is likely that there is some error correction along the largely task-redundant dimension of a target, particularly along the edges of the target. Thus, the error signal participants use along a task-redundant dimension remains unclear.

Classically, models of error-based learning correct to the center of the target [8, 10, 50–53]. However, when reaching towards a large target, it is unclear where an individual may aim. For example, if someone is throwing a ball into a large pool, they do not necessarily need to aim for the center of the pool. Rather, they could successfully throw the ball into the pool by aiming somewhere between the center and the edge of the pool.

**Fig 2:**
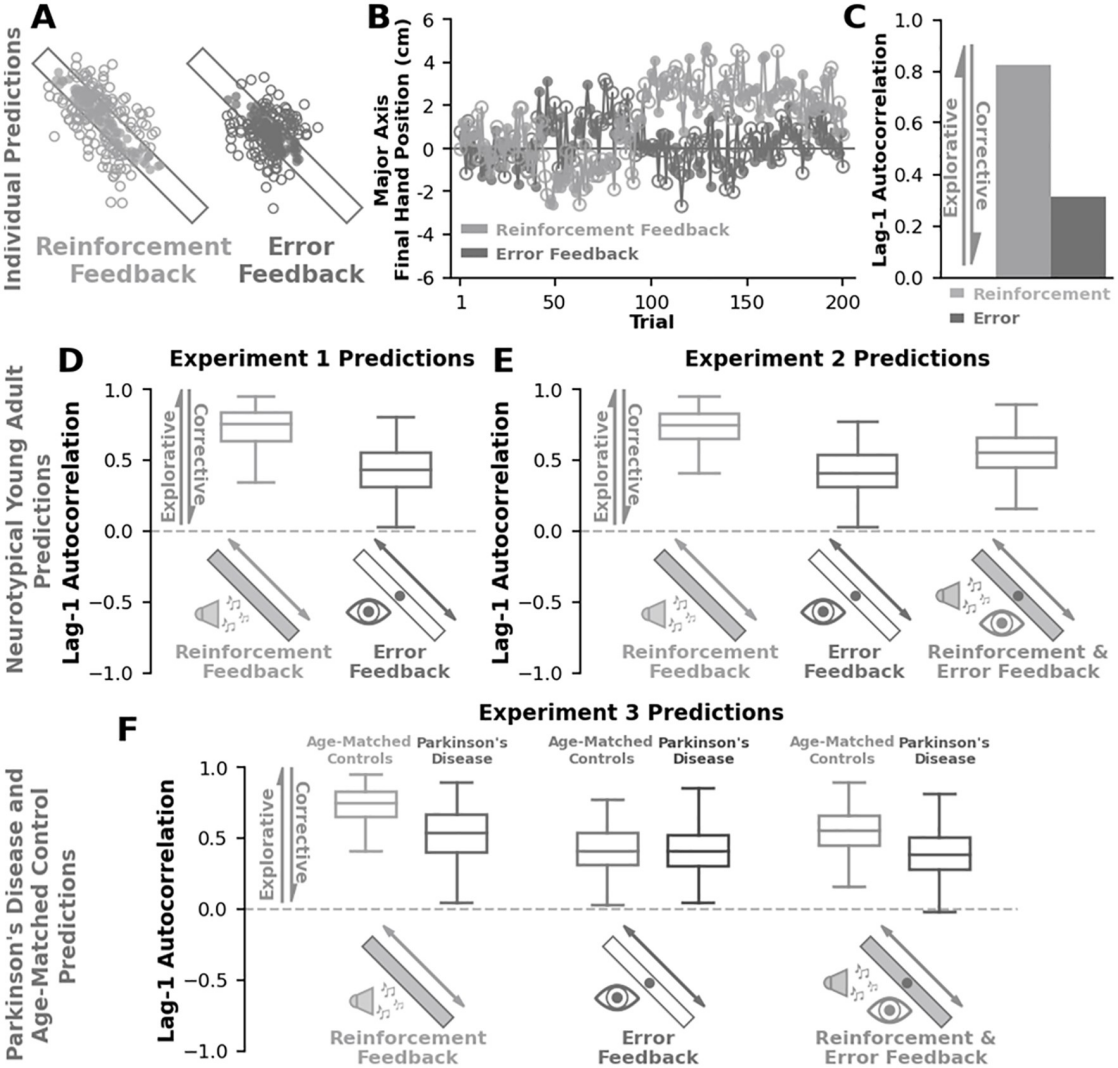
A priori model predictions. We generated theory-driven predictions for **Experiments 1–3**. Simulations of **A-C)** a single individual from **Experiment 1**. **D-F)** Group behaviour using our general model (Model 1). **A)** Final hand positions from a simulation of the reinforcement feedback (light grey) and error feedback (dark grey) conditions. Solid circles represent target hits while open circles represent target misses. **B)** Here we show the final hand positions along the major target axis (y-axis) for the reinforcement feedback (light grey) and error feedback (dark grey) conditions over trials (x-axis). **C)** When analyzing the lag-1 autocorrelation along the major axis, our model suggests greater lag-1 autocorrelation with reinforcement feedback (light grey) compared to error feedback (dark grey). Note that a higher lag-1 autocorrelation corresponds to greater exploration [3], whereas a lower lag-1 autocorrelation corresponds more with corrective actions [8]. We simulated 500 individuals for **D) Experiment 1**, **E) Experiment 2**, and **F) Experiment 3**. **D)** Here we show the lag-1 autocorrelation along the major target axis (y-axis) for each condition (x-axis) in **Experiment 1** (parameters: *σ*^*M*,*x*^ = 0.37, *σ*_*M*,*y*_ = 0.49, *σ*_*E*,*x*_ = 0.38, *σ*_*E*,*y*_ = 0.88, *α*_*x*_ = 0.99, *α*_*y*_ = 0.99, *β*^*aim*,*x*^ = 0.15, *β*^*target*,*x*^ = 0.13, *β*^*aim*,*y*^ = 0.25, *β*^*target*,*y*^ = 0.01). Our model suggests that reinforcement feedback (light grey) should yield greater lag-1 autocorrelations than error feedback (dark grey). Greater lag-1 autocorrelations with reinforcement feedback compared to error feedback would suggest that reinforcement-based processes boost exploration while error-based processes suppress exploration. **E)** Here we show the lag-1 autocorrelation along the major axis (y-axis) for each condition (x-axis) in **Experiment 2**. Using the same parameters our model suggests that reinforcement feedback (light grey) should yield the largest lag-1 autocorrelations, error feedback (dark grey) should yield the lowest lag-1 autocorrelations, and simultaneous reinforcement & error feedback should yield moderate lag-1 autocorrelations. A moderate level of lag-1 autocorrelations would suggest that reinforcement-based and error-based processes interact by mutually opposing one another during sensorimotor exploration. **F)** For **Experiment 3**, we modelled Parkinson’s disease participants (darker colours) by simply reducing the model parameter associated with reinforcement feedback (*α*_*x*_ = 0.5, *α*_*y*_ = 0.5). Age-matched control predictions (light colours) use the same model parameters as in **Experiment 1 & 2**. Our model would suggest that a deficit to reinforcement-based processes (Parkinson’s) should yield lower lag-1 autocorrelations in the reinforcement and reinforcement & error conditions compared to neurotypical age-matched controls. Box and whisker plots display the 25th, 50th, and 75th percentiles.

One potential error signal participants may use to update movement aim is the error between the executed movement and their intended movement [54, 55]. To investigate the error signal utilized along task-redundant dimensions, our general model considers both an error signal relative to the target center (*X*_*t*_ − *T*^*x*^) and an error signal to the intended movement aim ($X_{t}-X_{t}^{aim}$). As with our previous work [3], we modelled exploratory random walk behaviour with reinforcement feedback as a process of expanding movement variability after unsuccessful actions, and utilizing knowledge of this exploratory movement variability to update movement aim towards a recent success. Here, we developed a general model (Model 1) that considers both reinforcement-based and error-based processes. We used the general model to generate our *a priori* predictions (Fig 2). The general model simulates final hand position along the minor axis (*X*^*t*^) and major axis (*Y*^*t*^) as

$$X_{t}=X_{t}^{aim}+\epsilon_{t}^{M,x}+\left(1-r_{t-1}\right)\epsilon_{t}^{E,x}$$ (1A)

$$Y_{t}=Y_{t}^{aim}+\epsilon_{t}^{M,y}+\left(1-r_{t-1}\right)\epsilon_{t}^{E,y}$$ (1B)

$$X_{t+1}^{aim}=X_{t}^{aim}+r_{t}\alpha^{x}\left[\left(1-r_{t-1}\right)\epsilon_{t}^{E,x}\right]-\beta^{aim,x}\left(X_{t}-X_{t}^{aim}\right)-\beta^{target,x}\left(X_{t}-T^{x}\right)$$ (1C)

$$Y_{t+1}^{aim}=Y_{t}^{aim}+r_{t}\alpha^{y}\left[\left(1-r_{t-1}\right)\epsilon_{t}^{E,y}\right]-\beta^{aim,y}\left(Y_{t}-Y_{t}^{aim}\right)-\beta^{target,y}\left(Y_{t}-T^{y}\right)$$ (1D)

Final reach position on the current trial (*X*_*t*_, *Y*_*t*_) is equal to the intended movement aim ($X_{t}^{aim},Y_{t}^{aim}$) with additive Gaussian noise ($\epsilon_{t}^{i}∼N\left(0,\sigma_{i}^{2}\right)$). Superscripts represent the source of the variability: motor movement variability [56–58] (M) and exploratory movement variability (E). Exploratory movement variability is added only if the previous trial was unsuccessful [3–6] (*r*_*t*−1_ = 0). When given reinforcement feedback and the trial is successful (*r*_*t*_ = 1), movement aim is updated proportionally (*α*) to exploratory movement variability [3] if exploratory movement variability was present (*r*_*t*−1_ = 0). This reinforcement-based process of increasing exploratory movement variability following a failure and updating movement aim following a subsequent success boosts exploratory behaviour. For the general model, when provided error feedback, movement aim is partially corrected towards the intended movement aim [54] (*β*^*aim*^) and partially corrected towards the center of the target [8, 10, 11, 50–52] (*β*^*target*^). These error-based corrections to movement aim may suppress exploratory behaviour.

### Simulating individual behaviour

We used our general model (Model 1) to generate *a priori* predictions for all three experiments (Fig 2). Model parameters for the general model used to generate our *a priori* predictions were similar to our prior work on reinforcement-based random-walk behaviour and past work on error-based random-walk behaviour [3, 8]. Fig 2A shows a simulation of an individual in the reinforcement feedback condition and error feedback condition for **Experiment 1**. Fig 2B shows the component of the final hand position along the major target axis for both the reinforcement feedback condition and error feedback condition. In Fig 2C, we used lag-1 autocorrelation to quantify exploratory random walk behaviour along the major target axis for each condition. A higher lag-1 autocorrelation indicates greater exploration of the task-redundant dimension [3], while a lower lag-1 autocorrelation is associated with greater error corrections [8]. Here, lag-1 autocorrelations reflect the relative contribution of exploration and corrective actions along a continuum. For this simulated individual, we found a greater lag-1 autocorrelation in the reinforcement feedback condition compared to the error feedback condition.

### Simulating group behaviour

We used the general model (Model 1) to simulate 500 individuals in **Experiment 1** (Fig 2D), **Experiment 2** (Fig 2E), and **Experiment 3** (Fig 2F). The same set of model parameters were used to generate *a priori* predictions of neurotypical populations in **Experiment 1**, **Experiment 2**, and **Experiment 3** (Age-Matched Control Participants). Critically, in **Experiment 3** we wanted to capture how dysfunction in the basal ganglia, a brain region closely linked to reinforcement feedback, influences reinforcement-based exploration. Thus, we lowered the model parameter associated with reinforcement-based processes (*α*) to generate *a priori* predictions of participants with Parkinson’s disease. Lowering the parameter associated with reinforcement-based movement updates will decrease exploratory behaviour.

In **Experiment 1** (Fig 2D), our model predicts greater levels of lag-1 autocorrelation in the reinforcement feedback condition compared to the error feedback condition. In **Experiment 2** (Fig 2E), our model predicts the highest level of lag-1 autocorrelation in the reinforcement feedback condition, the lowest level of lag-1 autocorrelation in the error feedback condition. When given reinforcement & error feedback, our model predicts that lag-1 autocorrelations will be greater than when given error feedback but less than when given reinforcement feedback. This moderate level of lag-1 autocorrelation is a result of the reinforcement-based movement update and error-based correction acting in opposite directions. In **Experiment 3** (Fig 2F), our model predicts that participants with Parkinson’s disease will display lower levels of lag-1 autocorrelation compared to neurotypical age-matched controls for both the reinforcement feedback condition and the reinforcement & error feedback condition.

### Individual behaviour

**Fig 3:**
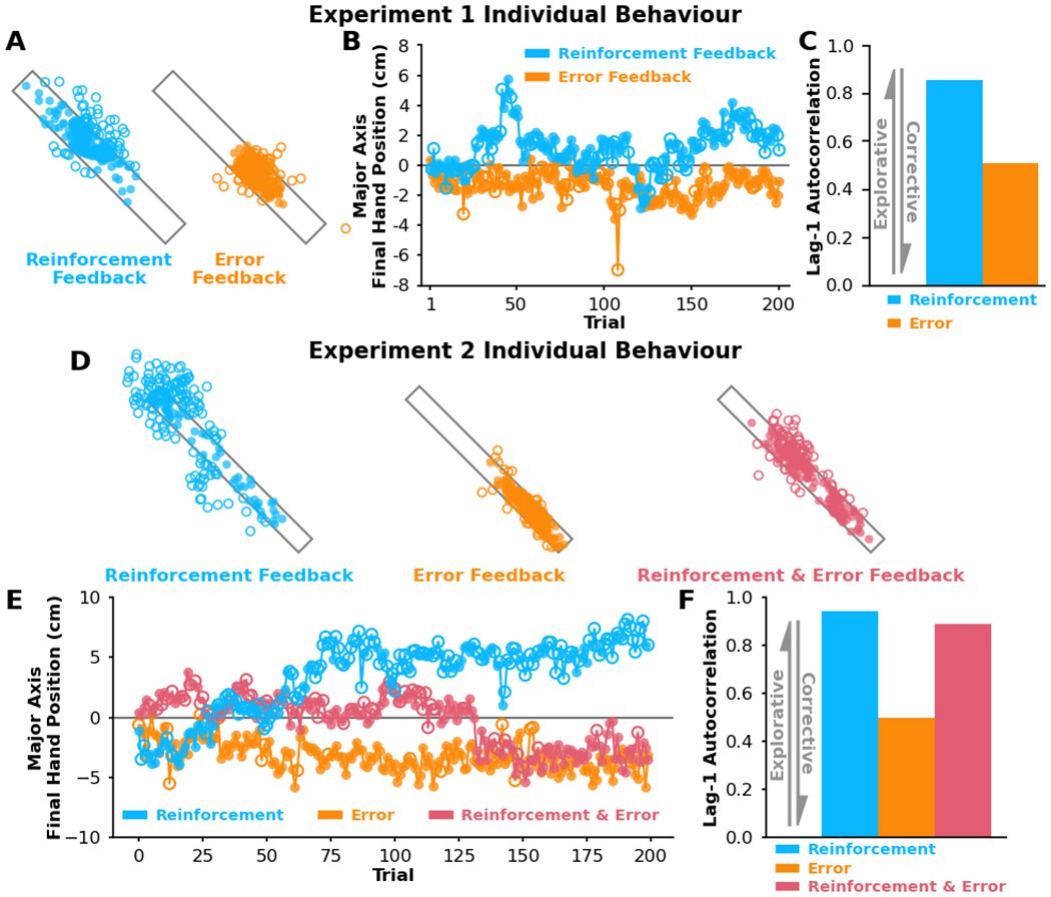
Individual behaviour for Experiment 1 and 2. **A)** Successful (solid circles) and unsuccessful (empty circles) final hand positions for a representative participant in **Experiment 1** that performed the reinforcement feedback condition (blue) and error feedback condition (orange). **B)** Final hand position along the major target axis (y-axis) for every trial (x-axis). **C)** We used lag-1 autocorrelation (y-axis) of final hand positions along the major axis to capture the level of exploration and corrective actions in each condition. Aligning with model predictions, this participant displayed greater lag-1 autocorrelation with reinforcement feedback (blue) compared to error feedback (orange). **D)** Successful (solid circles) and unsuccessful (empty circles) final hand positions for a representative participant in **Experiment 2** that performed the reinforcement feedback condition (blue), error feedback condition (orange), and reinforcement & error feedback condition (pink). **E)** Here we show the component of the final hand position along the major target axis (y-axis) for every trial (x-axis). **F)** Aligning with model predictions, this participant displayed the highest level of lag-1 autocorrelation with reinforcement feedback (blue), the lowest level of lag-1 autocorrelation with error feedback (orange), and an moderate level of lag-1 autocorrelation with reinforcement & error feedback (pink).

In **Experiment 1**, we investigated how reinforcement-based and error-based processes differentially influence exploration along task-redundant dimensions. Fig 3A shows final hand positions for a representative participant who experienced both the reinforcement feedback condition and error feedback condition. For this particular individual, we saw greater exploration along the major target axis with reinforcement feedback compared to error feedback (Fig 3B). We quantified this participant’s trial-by-trial exploration using lag-1 autocorrelation, where we saw greater lag-1 autocorrelations with reinforcement feedback compared to error feedback (Fig 3C).

### Group behaviour

**Fig 4:**
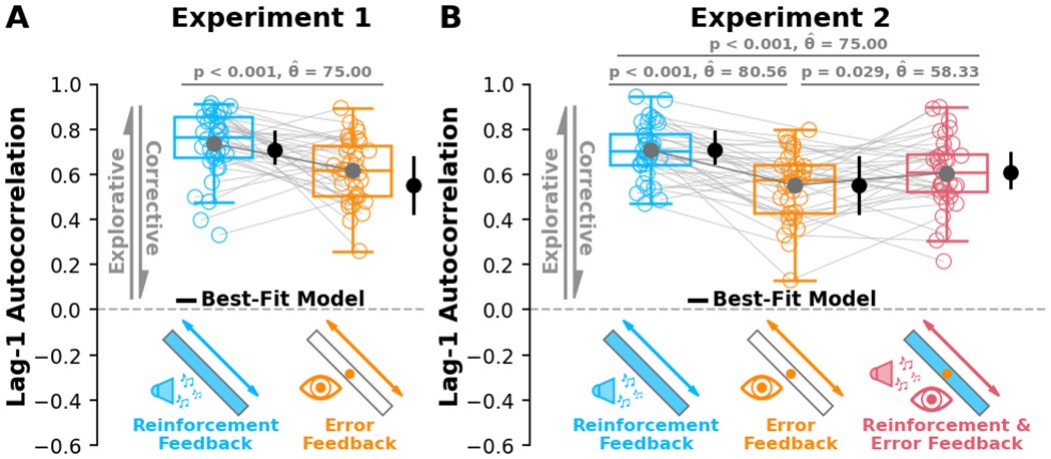
Group results for Experiment 1 and 2. **A)** Here we show lag-1 autocorrelation (y-axis) along the major target axis for each participant in both conditions (x-axis) in **Experiment 1**. Aligning with model predictions (Fig 2D), participants displayed significantly greater lag-1 autocorrelations in the reinforcement feedback condition (blue) compared to the error feedback condition (orange; p < 0.001). **B)** Lag-1 autocorrelation along the major target axis (y-axis) for each condition (x-axis) in **Experiment 2**. Replicating the results of **Experiment 1**, participants in the reinforcement feedback condition (blue) again displayed greater lag-1 autocorrelations than the error feedback condition (orange; p < 0.001). Interestingly, and aligning with model predictions (Fig 2E), participants receiving both reinforcement & error feedback simultaneously displayed greater lag-1 autocorrelations compared to just error feedback (p = 0.029) but less than just reinforcement feedback (p < 0.001). We performed a model comparison analysis to better understand the mechanism underlying sensorimotor exploration. Black solid circles and lines show the resulting mean and inner quartiles of the best-fit model simulations (Model 4) for both **A) Experiment 1** and **B) Experiment 2**. Box and whisker plots display the 25th, 50th, and 75th percentiles. Error bars on the best-fit model represent the 25th and 75th percentiles. Hollow circles and connecting lines represent individual data. Solid circles and connecting lines represent group means. Collectively, **Experiment 1** and 2 suggest that reinforcement feedback boosts exploration while error feedback suppresses exploration. Additionally, moderate levels of exploration were observed in the reinforcement & error feedback condition, supporting the idea that there exists an interaction between reinforcement-based and error-based processes in sensorimotor exploration.

Aligned with our group level *a priori* predictions (Fig 2D), we found significantly higher lag-1 autocorrelation in the reinforcement feedback condition compared to the error feedback condition (Fig 4A, p < 0.001, $\hat{\theta }=75.0$). Similarly, we found significantly greater lag-1 autocorrelation along the minor target axis during the reinforcement feedback condition compared to the error feedback condition (p < 0.001; see Fig A in S1 Appendix). Lag-1 autocorrelations along the minor axis appeared much lower than lag-1 autocorrelations along the major axis, suggesting more corrective actions along the minor axis. Additionally, we were able to replicate our **Experiment 1** results by re-analyzing a recent gait study [49] (see Fig C in S1 Appendix). These results suggest that reinforcement-based processes boost exploration by updating movement aim towards success while error-based processes suppress exploration aim by correcting movement aim in reaching and gait.

### Reinforcement-based and error-based processes interact to influence exploration during reaching individual behaviour

In **Experiment 2**, we wanted to replicate the results of **Experiment 1** while also investigating how reinforcement-based and error-based processes may interact during exploration of task-redundant dimensions. Fig 3D shows final hand positions for a representative participant who experienced the reinforcement feedback condition, error feedback condition, and reinforcement & error feedback condition. For this particular individual, we saw the greatest amount of exploration along the major target axis with reinforcement feedback, the lowest amount of exploration with error feedback, and a moderate amount of exploration with both reinforcement & error feedback (Fig 3E). Consequently, when quantifying exploration for this individual using lag-1 autocorrelation (Fig 3F), we saw the highest level of lag-1 autocorrelation with reinforcement feedback, the lowest level of lag-1 autocorrelation with error feedback, and a moderate level of lag-1 autocorrelation with reinforcement & error feedback.

### Group behaviour

Aligned with our group level *a priori* predictions (Fig 2E) and the results of **Experiment 1**, participants displayed significantly greater lag-1 autocorrelations along the major axis in the reinforcement feedback condition compared to the error feedback condition (Fig 4B; p < 0.001; $\hat{\theta }=80.56$). Likewise, when we analyzed lag-1 autocorrelation along the minor axis, participants displayed greater lag-1 autocorrelation during the reinforcement feedback condition compared to the error feedback condition (p < 0.001; see Fig A in S1 Appendix). These results replicate the findings of **Experiment 1** and further support the idea that reinforcement-based processes boost exploration while error-based processes suppress exploration.

Again aligning with *a priori* predictions, participants displayed greater lag-1 autocorrelations along the major axis in the reinforcement & error feedback condition compared to the error feedback condition (Fig 4B; p = 0.029; $\hat{\theta }=58.33$), but less than the reinforcement feedback condition (Fig 4B; p < 0.001; $\hat{\theta }=75.0$). Similarly along the minor axis, participants displayed greater lag-1 autocorrelations in the reinforcement & error feedback condition compared to the error feedback condition (p < 0.001; see Fig A in S1 Appendix), but less than the reinforcement feedback condition (p < 0.001; see Fig A in S1 Appendix). When acting in concert, our results suggest that reinforcement-based and error-based processes interact to result in moderate exploration.

### Dysfunction in the basal ganglia compromises reinforcement-based motor exploration individual behaviour

**Fig 5:**
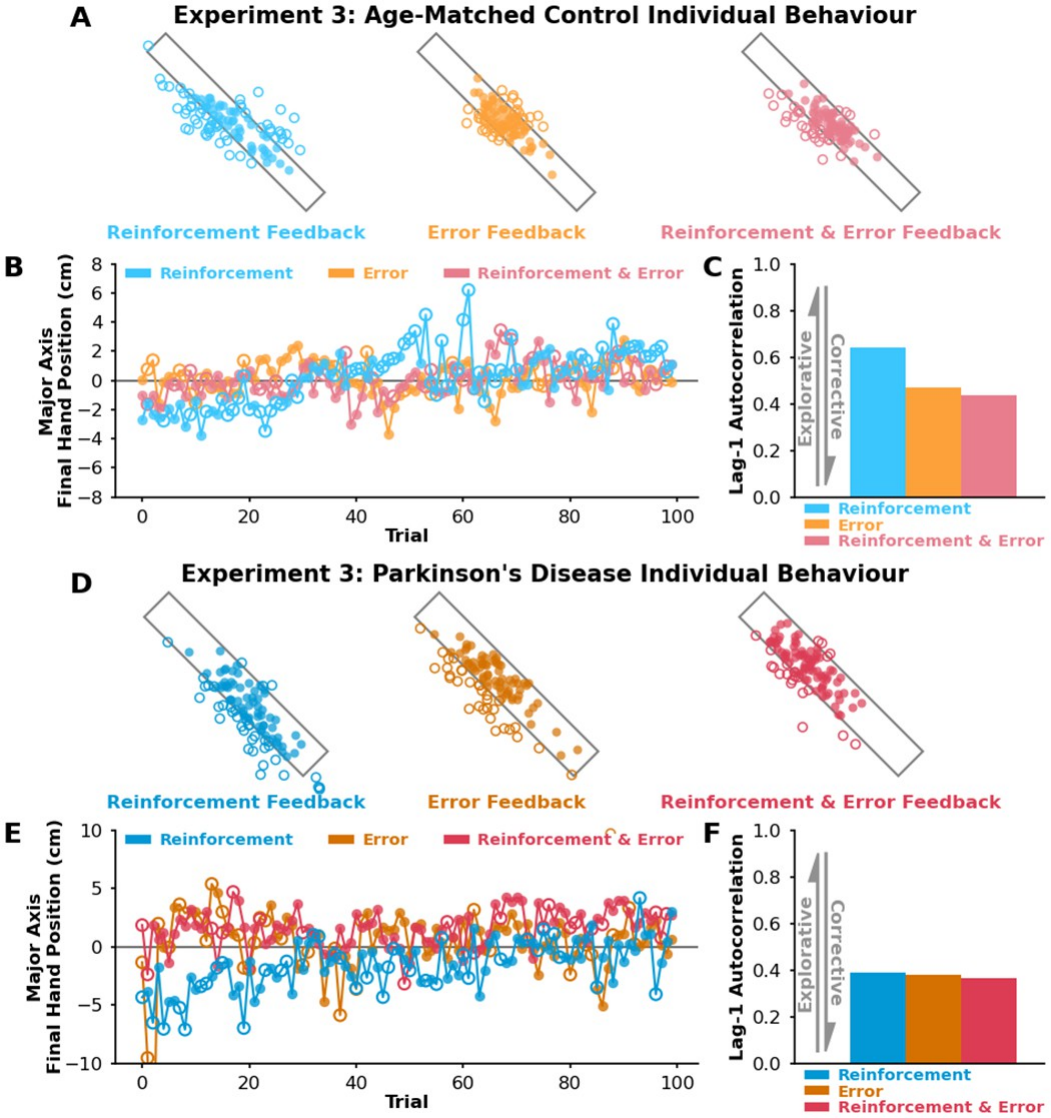
Experiment 3 age-matched control individual results. **A)** Successful (solid circles) and unsuccessful (empty circles) final hand positions for a representative age-matched control participant that performed the reinforcement feedback condition (light blue), error feedback condition (light orange), and reinforcement & error feedback condition (light pink). **B)** Here we show the component of the final hand position along the major target axis (y-axis) for every trial (x-axis). **C)** We used lag-1 autocorrelation (y-axis) of final hand positions along the major axis to capture the level of exploration in each condition. **D)** Successful (solid circles) and unsuccessful (empty circles) final hand positions for a representative participant with Parkinson’s disease that performed the reinforcement feedback condition (dark blue), error feedback condition (dark orange), and reinforcement & error feedback condition (dark pink). **E)** Here we show the component of the final hand position along the major target axis (y-axis) for every trial (x-axis). **F)** This representative participant with Parkinson’s disease did not display a change in lag-1 autocorrelation between conditions, suggesting that the basal ganglia influences sensorimotor exploration.

Exploratory behaviour in songbirds has been linked to reinforcement and the basal ganglia [15–17]. By studying those with Parkinson’s disease, the basal ganglia has been implicated in the modulation of exploratory movement variability in response to reinforcement feedback [6]. In **Experiment 3**, we wanted to gain mechanistic insight into the role of basal ganglia and associated reinforcement (reward) pathways in the exploration of task-redundant dimensions. We recruited participants with Parkinson’s disease as a population with known dysfunction in the basal ganglia. We also recruited a neurotypical age-matched control group. There was no statistical difference in age between the Parkinson’s disease group and the neurotypical age-matched control group (p = 0.69). All participants with Parkinson’s disease were on their routine dopaminergic medication during the study. Table 1 shows the Unified Parkinson’s Disease Rating Scale (UPDRS) results of each participant with Parkinson’s disease, which is used to rate the severity of symptoms. Both the Parkinson’s disease group and the neurotypical age-matched control group performed the Mini-Mental State Exam to rule out cognitive impairments such as dementia [59]. All participants scored higher than 26 on the exam, and we found no statistical difference in scores between the Parkinson’s disease group and the neurotypical age-matched control group (p = 0.82). Fig 5A shows final hand positions for a representative age-matched control participant who experienced the reinforcement feedback condition, error feedback condition, and reinforcement & error feedback condition. For this particular individual, we saw the greatest amount of exploration along the major target axis in the reinforcement feedback condition (Fig 5B). When quantifying exploration for this individual using lag-1 autocorrelation (Fig 5C), we saw the highest level of lag-1 autocorrelation in the reinforcement feedback condition.

**Table 1:** UPDRS Scores for Parkinson’s participants. We administered the Universal Parkinson’s Disease Rating Scale (UPDRS) to each participant in the Parkinson’s disease group of Experiment 3. Here we show individual section scores of the UPDRS for each participant. Higher scores indicate greater impairment. Section 1 of the UPDRS rates the non-motor aspects of daily living. Section 2 of the UPDRS rates the motor aspects of daily living. Section 3 of the UPDRS rates the severity of motor symptoms for each individual with Parkinson’s disease. Section 4 of the UPDRS rates motor complications.

Participant	1	2	3	4	5	6	7	8	9	10
Age (years)	68	72	63	51	68	61	62	82	72	60
Disease Duration (years)	12	6	1	3	6	6	6	4	1	4
Section 1	3	6	8	5	10	13	12	7	5	4
Section 2	3	3	10	3	7	10	24	5	2	12
Section 3	17	24	29	38	40	34	34	26	17	14
Section 4	2	6	5	3	8	1	1	3	0	0
Hoehn-Yahr Stage	2	2	2	2	2	2	2	2	2	2
MMSE	30	30	27	30	28	30	30	30	29	29

Fig 5D shows final hand positions for a representative participant with Parkinson’s disease who experienced the reinforcement feedback condition, error feedback condition, and reinforcement & error feedback condition. For this particular individual with Parkinson’s disease, we saw the same relative amount of exploration across all three conditions (Fig 5E). We did not see modulation of lag-1 autocorrelation (Fig 5F) between conditions for this representative participant with Parkinson’s disease.

### Group behaviour

**Fig 6:**
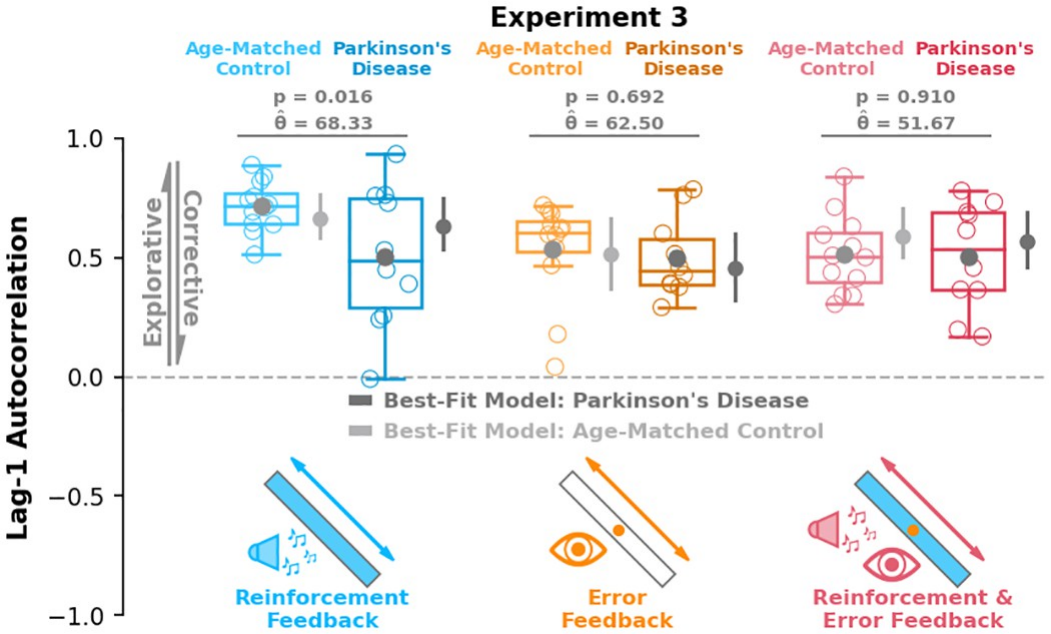
Parkinson’s and age-matched control results. Here we show lag-1 autocorrelation (y-axis) in each condition (x-axis), for both the neurotypical age-matched control (light colors) and Parkinson’s disease (dark colors) groups. Aligning with model predictions (Fig 2F), participants with Parkinson’s disease displayed significantly lower lag-1 autocorrelations compared to age-matched control participants in the reinforcement feedback condition (p = 0.016). Participants with Parkinson’s disease displayed similar levels of lag-1 autocorrelation compared to age-matched controls in the error feedback (p = 0.693) and reinforcement & error feedback (p = 0.910) conditions. We fit the results of both the Parkinson’s disease group (dark grey) and age-matched control group (light grey) separately using our best-fit model (Model 4), which found a reduced parameter value associated with reinforcement-based aiming updates in Parkinson’s disease. Taken together, our modeling and behavioural results suggest that the basal ganglia is involved with reinforcement-based task exploration. Box and whisker plots display the 25th, 50th, and 75th percentiles. Error bars on the best-fit model represent the 25th and 75th percentiles. Hollow circles represent individual data. Solid circles represent group means.

Aligning with *a priori* model predictions (Fig 2F), participants with Parkinson’s disease displayed significantly lower lag-1 autocorrelations in the reinforcement feedback condition compared to age-matched control participants (Fig 6; p = 0.016, $\hat{\theta }=68.33$). These results suggest that dysfunction in the basal ganglia compromises reinforcement-based sensorimotor exploration of task-redundant dimensions. Further matching *a priori* predictions, we did not find differences in lag-1 autocorrelation between Parkinson’s disease and age-matched control participants in either the error feedback condition (Fig 6, p = 0.693, $\hat{\theta }=62.5$). This was expected because Parkinson’s disease is not associated with error-based neural processes. We did not find a difference between groups in the reinforcement & error feedback condition (Fig 6, p = 0.91, $\hat{\theta }=51.67$), which was unexpected but may be due to a relatively weaker influence of reinforcement-based processes in this condition. We did not see significant differences between group lag-1 autocorrelations along the minor target axis (see Fig B in S1 Appendix).

Aligning with a priori model predictions (Fig 2F), participants with Parkinson’s disease did not display modulation of lag-1 autocorrelations across conditions (p > 0.94 for all comparisons). Matching a priori predictions, participants in the age-matched control group displayed greater lag-1 autocorrelations in the reinforcement feedback condition compared to the error feedback condition (p < 0.01) and the reinforcement & error feedback condition (p < 0.01). Unlike our a priori predictions and findings in **Experiment 2**, we did not see a difference in lag-1 autocorrelations between the error feedback and reinforcement & error feedback condition in the age-matched control group (p = 0.59). While unexpected, no difference in lag-1 autocorrelations between the error feedback and reinforcement & error feedback conditions in the age-matched control group may be due to a relatively weaker influence of reinforcement-based processes with age [60, 61].

In addition to lag-1 autocorrelation, past work has shown that trial-by-trial movement variability can be modulated by task success [3–6, 18, 23, 24, 62, 63]. Aligning with past work, participants in **Experiment 1** and **Experiment 2** displayed significantly greater movement variability following a target miss along both the minor and major target axes (p < 0.001 for all comparisons, see Fig D in S1 Appendix). In **Experiment 3**, the age-matched control group displayed significantly greater movement variability following a target miss compared to a target hit along both the minor and major axes (p ≤ 0.027 for all comparisons, see Fig D in S1 Appendix). In the group with Parkinson’s disease during **Experiment 3**, participants displayed significantly greater movement variability following a target miss compared to a target hit in all conditions along the minor axis (p < 0.001 for all comparisons, see Fig D in S1 Appendix) as well as along the major axis during the reinforcement & error feedback condition (p < 0.001, see Fig E in S1 Appendix).

**Fig 7:**
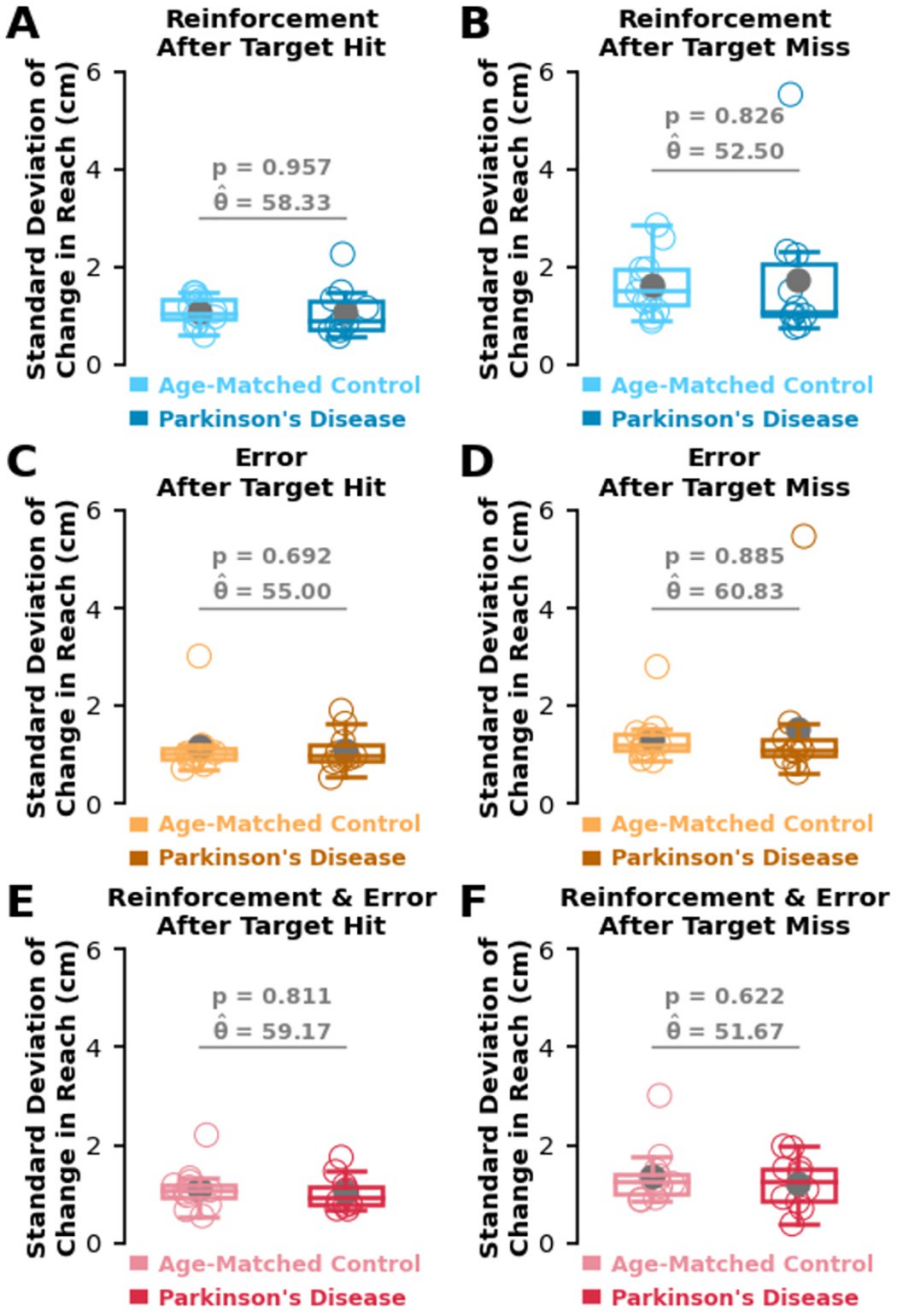
Movement variability conditioned on task outcome. **A-F)** We calculated trial-by-trial movement variability as the standard deviation of change in reach (y-axis) following target hits (left column) and target misses (right column) for both the age-matched control group (light colours) and Parkinson’s disease group (dark colours). Unlike past work [6], we did not find a significant difference between the age-matched control group and the Parkinson’s disease group for movement variability following a target hit or a target miss in any of the three conditions. These results suggest that Parkinson’s disease did not reduce trial-by-trial movement variability. Box and whisker plots display the 25th, 50th, and 75th percentiles. Hollow circles represent individual data. Solid circles represent group means.

Additionally, Parkinson’s disease has been shown to decrease movement variability after unsuccessful movements compared to neurotypical age-matched control participants [6]. Surprisingly, we did not find evidence that Parkinson’s disease reduced movement variability compared to age-matched control participants following either a successful reach or an unsuccessful reach in any experimental condition (Fig 7). We also did not find a difference in movement variability between participants with Parkinson’s disease and age-matched control participants along the minor target axis (see Fig F in S1 Appendix). These results suggest that Parkinson’s disease does not reduce the exploratory movement variability following an unsuccessful action. Rather, these findings suggest a reduced ability to utilize exploratory movement variability to update movement aim following a successful action.

### Best-fit model

Our general model (Model 1) updates trial-by-trial movement aim by considering both error-based and reinforcement-based processes. This general model did well to formulate *a priori* theory-driven predictions. However, models with many free parameters can be prone to overfitting to the data, and it is possible that one or more of the free parameters used by the model are not needed to explain our findings. Thus, we performed a model reduction analysis [3] to find the simplest model that would best explain the data using the fewest number of free parameters. We systematically reduced the parameters in the general model (Model 1) to test different mechanisms underlying sensorimotor exploration. Our prior work showed that reinforcement-based processes expand movement variability following an unsuccessful action. Knowledge of this movement.

Each model followed the same fitting procedure. Specifically, each model was fit to average participant lag-1 autocorrelations simultaneously across all experiments (see Methods). Model simulations for each fitted model can be found in Fig G in S1 Appendix. We used both Bayesian Information Criteria (BIC) and Akaike Information Criteria (AIC) analyses to weigh how well each model fit the data while penalizing the number of free parameters. A lower score indicates a better model fit for both BIC and AIC analyses. Both BIC and AIC analyses support Model 4 as the best-fit model (Table 2). Model 4 simulates final reach position and updates intended movement aim as

$$\begin{array}{c} X_{t}=X_{t}^{aim}+\epsilon_{t}^{M,x}+\left(1-r_{t-1}\right)\epsilon_{t}^{E,x} \end{array}$$ (4A)

$$\begin{array}{c} Y_{t}=Y_{t}^{aim}+\epsilon_{t}^{M,y}+\left(1-r_{t-1}\right)\epsilon_{t}^{E,y} \end{array}$$ (4B)

$$\begin{array}{c} X_{t+1}^{aim}=X_{t}^{aim}+r_{t}\alpha^{x}\left[\left(1-r_{t-1}\right)\epsilon_{t}^{E,x}\right]-\beta^{aim,x}\left(X_{t}-X_{t}^{aim}\right)-\beta^{target,x}\left(X_{t}-T^{x}\right) \end{array}$$ (4C)

$$\begin{array}{c} Y_{t+1}^{aim}=Y_{t}^{aim}+r_{t}\alpha^{y}\left[\left(1-r_{t-1}\right)\epsilon_{t}^{E,y}\right]-\beta^{aim,y}\left(Y_{t}-Y_{t}^{aim}\right) \end{array}$$ (4D)

**Table 2:** Model selection analysis. We performed a model selection analysis using both Akaike Information Criteria (AIC) and Bayesian Information Criteria (BIC). Both AIC and BIC consider how well a model fits the data while penalizing the number of free parameters. For both analyses, a lower score indicates a better model fit given the total number of free parameters. Experiments 1 & 2 were fit simultaneously and given a single combined score for both AIC and BIC. For Experiment 3, the Parkinson’s disease and age-matched control groups were fit separately and each group was given their own AIC and BIC scores. The table shows the sum of all AIC and BIC scores calculated for Experiments 1, 2, & 3. Both analyses would suggest Model 4 as the best-fit model across all experiments.

	Number of Parameters	Parameters Removed	Total Loss	AIC Score	BIC Score
Model 1	10	None	0.19	-0.39	-0.28
Model 2	8	*β*^*target*,*x*^, *β*^*target*,*y*^	0.82	17.42	17.76
Model 3	8	*β*^*aim*,*x*^, *β*^*aim*,*y*^	2.67	40.43	40.77
Model 4	9	*β* ^*target*,*y*^	0.23	-5.31	-5.09
Model 5	9	*β* ^*aim*,*y*^	2.53	45.69	45.92
Model 6	9	*β* ^*aim*,*x*^	0.33	4.60	4.82
Model 7	9	*β* ^*target*,*x*^	0.78	23.51	23.74
Model 8	8	*β*^*target*,*x*^, *β*^*aim*,*y*^	3.10	42.64	42.98
Model 9	8	*β*^*aim*,*x*^, *β*^*target*,*y*^	0.36	-1.80	-1.46

Unlike the general model (Model 1), the best-fit model (Model 4) does not update intended movement aim based on error corrections to both the target center and intended movement aim simultaneously in both dimensions. Rather, the best-fit model (Model 4) only considers both corrections to the target center and intended movement aim along the task-relevant dimension (minor target axis) and only corrections towards the intended movement aim along the task-redundant dimension (major target axis). Thus, our best-fit model (Model 4) does not make corrections towards the target center along the task-redundant dimension. Rather our best-fit model (Model 4) allows for a correction to somewhere between the center and edge of the target, similar to our prior example of throwing a ball into a large pool.

We obtained posterior parameter distribution estimates of the model parameters (see Fig I in S1 Appendix). We used the median values of the parameter distribution estimates to simulate each experiment. Model simulations from the best-fit model are shown alongside the participant data for **Experiment 1** (Fig 4A), **Experiment 2** (Fig 4B), Wood and colleagues (2024; see Fig C in S1 Appendix), and **Experiment 3** (Fig 7). The best-fit model did well to capture the trends in each experiment across both the major and minor axes (see Fig G in S1 Appendix).

The parameter associated with reinforcement-based processes along the task-redundant dimension was found to be lower in Parkinson’s disease (*α*^*y*^ = 0.52) compared to neurotypical controls (*α*^*y*^ = 0.79) that, aligned with model 1, predicted slightly less exploratory behaviour in the reinforcement feedback condition. These results suggest that Parkinson’s disease reduces the ability to utilize exploratory variability to update movement aim towards successful actions.

Taken together, our results and modelling suggest that reinforcement-based processes boost exploratory behaviour by updating movement aim towards a success, while error-based processes suppress exploratory behaviour by correcting movement aim. Our modelling analysis suggests that reinforcement-based processes and error-based processes interact by mutually opposing one another, leading to moderate levels of exploration. Additionally, our results from **Experiment 3** suggest that Parkinson’s disease does not reduce the exploratory movement variability following an unsuccessful action. Rather, our results suggest that Parkinson’s disease reduces the ability to utilize exploratory movement variability to update movement aim following a successful action.

## Discussion

Here we show that reinforcement-based and error-based processes differentially influence sensorimotor exploration along task-redundant dimensions. Specifically, reinforcement based processes promote exploratory behaviour by updating movement aim towards a recent success, while error-based processes suppress exploratory behaviour through error-based corrections to movement aim. Our empirical and modelling results suggest that reinforcement-based and error-based processes interact by mutually opposing one another, leading to moderate levels of sensorimotor exploration. Our results with Parkinson’s disease suggest that dysfunction in the basal ganglia reduces the ability to utilize exploratory movement variability when updating movement aim, leading to less exploration.

An important goal of **Experiment 2** was to test the idea that reinforcement-based and error-based processes interact to influence sensorimotor behaviour. Crucially, if these processes interact, we would expect the reinforcement & error condition to display less than the reinforcement condition and greater exploration than the error condition. As simulated by our *a priori* model (Fig 2E) and best-fit model, we found that the reinforcement & error condition had respectively less and greater exploration compared to the reinforcement condition and error condition (Fig 4B). Past work has suggested that an intrinsic reinforcement-based signal may be present following a successful movement when given only error-based feedback [10]. It can be difficult to completely isolate error-based feedback from reinforcement-based feedback. However, in our paradigm, participants are provided an extrinsic reward in the reinforcement feedback condition and reinforcement & error feedback condition, which has been shown to boost dopaminergic activity [64] and motor learning [65]. Thus, while not completely isolating error-based feedback from intrinsic reinforcement-based feedback, our paradigm is able to distinguish error-based feedback from extrinsic reinforcement-based feedback. Nevertheless, these results are in line with converging anatomical evidence of neural connections between the basal ganglia and cerebellum [40–43], and support the hypothesis that reinforcement-based and error-based processes interact to influence sensorimotor exploration.

The best-fit model (Model 4) would suggest that this moderate level of exploration arises from reinforcement-based processes and error-based processes acting in opposite directions. Along the task-redundant dimension, the best-fit model locally updates movement aim towards a recent success by utilizing knowledge of exploratory movement variability. In addition to this reinforcement-based update, the best-fit model also corrects movement aim so that the executed movement more closely resembles the previously intended movement. Thus, the reinforcement-based movement update pushes movement aim towards a recent success while the error-based correction pulls towards the previously intended movement. The net result of this push and pull respectively by reinforcement-based and error-based mechanisms leads to a moderate level of exploratory random walk behaviour. This moderate level of exploratory random walk behaviour is lower compared to isolated reinforcement-based mechanisms and greater compared to isolated error-based mechanisms. As currently formulated, both reinforcement-based and error-based mechanisms in our model individually update intended movement aim. For instance, reinforcement-based processes may update movement aim on one trial, and this updated movement aim will be used as part of the error signal on the next trial. Thus, over multiple trials these individual updates from reinforcement-based and error-based mechanisms begin to indirectly interact with one another. Our past work showed dissociable influences of reinforcement-based and error-based processes during sensorimotor adaptation [48]. We also found that when reinforcement-based and error-based processes have different optimal solutions given externally provided noise, that the sensorimotor system will suppress the influence of reinforcement feedback. However, for the task used in this paper, reinforcement-based and error-based processes both reach towards previous movement locations that have been successful. Here we show that reinforcement feedback and error feedback indirectly interact with one another when both are provided in a complementary manner.

Our model suggests reinforcement-based and error-based processes indirectly interact to influence exploration. This framework is analogous to how the basal ganglia and cerebellum share interconnections to the same motor planning circuitry such as the dorsal premotor [44] and prefrontal cortices [45]. However, it is also possible that reinforcement-based and error-based processes may directly influence each other as the basal ganglia and cerebellum also share direct connections [40–42]. One possible mechanism for a direct interaction between reinforcement-based and error-based processes may be through the history of reinforcement (i.e., reward). Importantly, the history of reinforcement influences both expected reward and reward prediction error. Expected reward can be thought of as a weighted, running average of reward received from previous trials. Expected reward has been shown to play an important role in cognitive decision making tasks [66, 67]. Reward prediction error is the difference between expected reward and received reward for an action. Reward prediction errors promote neuroplasticity [54, 68–70] and are used to recursively update expected reward [71]. The cerebellum is typically associated with error-based processes [27–31]. However, recent work has identified reward signatures in the cerebellum that encode expected reward [46, 47] and reward prediction error [72, 73] Thus, it is possible that reinforcement-based processes directly interact with error-based processes through expected value or reward prediction errors. Future work should examine how reinforcement history, specifically through expected reward or reward prediction errors, influence error-based corrective behaviour.

Our findings suggest that the sensorimotor system continually explores by greedily updating reach aim towards the last successful movement, with error corrections to their previous aim somewhat dampening this exploration. Such a process is a reasonable general exploration strategy across a wide range of environments to maximize reward [4]. This process was likely occurring in our task that was designed to isolate exploratory processes [18], even though there was likely minimal value in continually updating reach aim along the task-redundant dimension. Although it is possible that participants were to some degree exploring in an attempt to minimize energy [33] or sensorimotor noise [74], which to some extent may have influenced their reach aim. Nevertheless here we show that reinforcement boosts exploration along a task-redundant dimension in task space, which we posit may also occur along redundant dimensions in muscle and joint space. Our work differs from the Tolerance, Noise, Covariation (TNC) approach (task space) [74, 75], the uncontrolled manifold hypothesis (joint space) [32], and minimum intervention principal from optimal feedback control (task space [76], joint space [33], muscle space [77]), all of which do not consider the role of reinforcement processes influencing movement variability along task-redundant dimensions. Optimal feedback control explains greater movement variability along task-redundant dimensions to arise by not making corrective movements along this dimension because it is energetically costly. Conversely, our past work showed that greater movement variability along task-redundant dimensions can arise through reinforcement-based processes continually updating reach aim towards recently successful actions [3]. In the present study, our results suggest that error-based processes are still to some degree involved with corrective actions towards recently successful aim locations, which influence movement variability along the task redundant dimension.

In **Experiment 3**, we recruited participants with Parkinson’s disease to gain mechanistic insight into the role of the basal ganglia in sensorimotor exploration. Aligning with *a priori* predictions (Fig 2F), we found that Parkinson’s disease reduced reinforcement-based exploratory random walk behaviour compared to neurotypical age-matched controls. Thus, our results suggest that a compromise to the basal ganglia compromises exploratory random walk behaviour. Contrary to previous findings [6], this decrease in exploratory behaviour was only found in the exploratory random walk behaviour and not the magnitude of movement variability following an unsuccessful action. Our behavioural data, *a priori* model, and best-fit model suggest this difference arises in part due to a decrease in the knowledge of exploratory variability (*α*) used to update movement aim. That is, although participants with Parkinson’s disease modulated movement variability between successful and unsuccessful trials, they were less able to use knowledge of this exploratory movement variability to update movement aim following rewarded reaches. The best fit model predicted these behavioural differences, albeit they were less pronounced than the *a priori* model. Notably, we did not find statistical differences in exploratory random walk behaviour between Parkinson’s disease and age-matched control participants in the error feedback or reinforcement & error feedback conditions. As in past work [78–80], the similarity between the Parkinson’s disease group and age-matched control group when given error feedback suggests that error-based circuitry driving sensorimotor exploration remains intact with Parkinson’s disease. Our results suggest that reinforcement-based processes impact exploratory random walk behaviour, which may be exploited to discover new and successful motor actions in neurological conditions where the basal ganglia and associated reinforcement (reward) pathways remain intact.

We found no difference between the error feedback and reinforcement & error feedback conditions in the age-matched control group of **Experiment 3**. One possible explanation is that the interaction between reinforcement-based and error-based processes weakens with age. In rat models, recent work has shown that the cerebellum has a direct connection to the substantia nigra [43], an area that is known to degrade with age in humans [60, 61]. It is possible that this direct connection between the basal ganglia and cerebellum naturally degrades with age, resulting in a weaker interaction between reinforcement-based and error-based neural circuits. It would be useful for future work to further examine how neural circuits change over time from childhood through adulthood.

Similar to past work [6], participants with Parkinson’s disease performed **Experiment 3** while on their routine dopaminergic medication. While we used a Parkinson’s disease sample size similar to past work [6], this small sample size of individuals with Parkinson’s disease that had diverse symptoms is a limitation of our work. Additionally, having participants on their routine medication allowed us to observe behaviour during the participant’s normal functioning state. Observing behaviour while in the on-medication state likely improved the ability of participants with Parkinson’s disease to complete the motor components of the task. However, as a result, it can be difficult to parse the effects of Parkinson’s disease from the effects of the dopaminergic medication. Indeed, some dopaminergic medications have been found to alter learning during reward-based tasks in neurotypical populations [81, 82]. It has been proposed that individuals with Parkinson’s disease on dopaminergic medication can display decreased task performance as a result of dopamine overdose [83]. According to the dopamine overdose hypothesis, reinforcement-based neural processes become compromised due to overstimulation of the dopaminergic system [83]. Overstimulation of the dopaminergic system leads to a lack of responsiveness to reinforcement feedback. Thus, it is possible that either dysfunction of the basal ganglia and or dopamine overdose could have led to decreased exploratory behaviour in our task. Nevertheless, either dysfunction of the basal ganglia or dopamine overdose represent a compromised reinforcement-based process. Moving forward, it would be beneficial to study the influence of on-medication and off-medication states in Parkinson’s disease to isolate the influence of dopamine overdose or basal ganglia function on exploratory behaviour.

Our best-fit model (Model 4) makes corrections towards both the target center and intended movement aim along the task-relevant dimension (minor axis) and only corrections towards the intended movement along the task-redundant dimension (major axis). Intended movement aim has been shown to play a critical role in sensorimotor adaptation [55, 84]. Work by McDougle and colleagues (2017) investigate generalization during a visuomotor rotation task. They found evidence to suggest that the intended movement goal is actively corrected towards by the sensorimotor system during motor adaptation. Their findings complement our work, where the models support the idea that individuals will make corrective actions towards previously intended movement aim. However, in our task it would be difficult to distinguish between the intended movement aim and the center of the target along the task-relevant dimension due to the small width of the target. It would be interesting to further examine how error signals are weighted along different dimensions of a task, possibly by including aiming reports [3, 11, 55, 85] or experimentally imposing an explicit strategy [9].

Van Beers and colleagues (2013) provide evidence to suggest that this error signal is not present along the task-redundant dimension of a task. Specifically, they had participants reach towards a long thin target. Participants displayed greater exploratory random walk behaviour along the task-redundant dimension compared to the task-relevant dimension. Van Beers and colleagues (2013) suggested that greater exploratory random walk behaviour along the task-redundant dimension arose due to the lack of an error signal relative to the target and the accumulation of planned movement variability. However, without an error signal, the accumulation of planned movement variability would eventually cause the executed movement to be off the target. It is likely that some form of error signal remains along the task-redundant dimension, particularly along the edges of the target. As previously mentioned, one does not need to aim for the center when throwing a ball into a large pool. Rather, aiming to a point between the center and edge of the pool would also produce a successful movement. This raises a simple question: where do participants aim on a large target? Thus, as part of our modelling work, we investigated the error signal used by the sensorimotor system to make corrections along task-redundant dimensions.

The results of our best-fit model (Model 4) suggest that the error signal utilized along the task-redundant dimension is the difference between the executed reach and the previously intended movement. While conceptually different, utilizing the difference between the executed reach and the previously intended movement as an error signal is mathematically similar to the van Beers and colleagues (2013) model (see S1 Appendix). Specifically, in both models a portion of the movement variability that produces the executed movement is used to update the intended movement of the subsequent trial. A notable difference between our best-fit model (Model 4) and the model of van Beers and colleagues (2013) is that our model does not use planned movement variability that constantly accumulates, but instead uses exploratory movement variability conditioned on success. Planned movement variability results from the noise arising in the premotor cortex during the planning stages of movement [38, 86], which results in the same planned movement being executed slightly differently on different trials. As mentioned in our prior work [3], it is unclear whether planned movement variability arising from the premotor cortex [38, 39] and exploratory movement variability arising from the basal ganglia [15, 16, 87] are unrelated processes. Indeed, the premotor cortex and basal ganglia are known to be linked through a neural loop [88, 89]. Thus, it would be useful for future work to further investigate how motor, planned, and exploratory movement variability individually contribute to sensorimotor behaviour.

Here we investigated the roles and interplay between reinforcement-based processes and error-based processes on sensorimotor exploration. Across three reaching experiments and a gait study [49], we found evidence that reinforcement-based processes update movement aim towards a success while error-based processes correct movement aim towards the intended movement. We also found when acting in concert that these reinforcement-based and error-based processes interact by opposing one another and cause moderate levels of exploratory random walk behaviour. Our findings for those with Parkinson’s disease found less exploratory random walk behaviour but, unlike previous work [6], no changes in exploratory movement variability following a failure. Thus, Parkinson’s disease may have led to reduced knowledge of exploratory movement variability that is used to update movement aim. Understanding the individual and interacting processes underpinning sensorimotor exploration may lead to the development of better informed neurorehabilitation paradigms that aim to discover new and successful functional motor skills during recovery [1, 2, 13, 14].

## Methods

### Ethics statement

Each participant provided written informed consent to procedures approved by the University of Delaware’s International Review Board.

### Participants

Across all three experiments we recruited 94 participants. We recruited 72 young neurotypical participants across **Experiment 1** (n = 36, 20.4 years ± 2.7 SD) and **Experiment 2** (n = 36, 20.6 years ± 2.2 SD). Participants reported they were right-handed and free of neuromuscular disease.

In **Experiment 3**, we collected data from participants with Parkinson’s disease (n = 10, 69.7 years ± 6.9 SD) and neurotypical age-matched controls (n = 12, 68.4 years ± 8.4 SD). All participants with Parkinson’s disease and neurotypical age-matched controls were free of dementia as assessed by the Mini-Mental State Exam [59] (MMSE score > 26). All participants were free of neurological disease other than Parkinson’s disease. Participants with Parkinson’s disease were on their normal dopaminergic medication at the time of testing [6], consistent with their normal functioning state. Two members of the research team, including an occupational therapist, jointly scored disease severity using the Unified Parkinson’s Disease Rating Scale [90] (UPDRS). The results of the screening are reported in Table 1.

### Apparatus

Participants grasped the handle of a robotic manipulandum (Fig 1A, KINARM, BKIN Technologies, Kingston, ON, Canada) and made reaching movements in the horizontal plane. A semi-silvered mirror blocked vision of both the participant’s upper-limb and the robotic arm, and projected images (start position, target) from an LCD screen onto the horizontal plane of motion. Hand position was recorded at 1000 Hz and stored offline for data analysis.

### General task protocol

Participants were presented with virtual images of a start position (white circle, radius = 0.75cm) and a target. The start position was aligned with the sagittal plane and approximately 15 cm away from their body. Displayed targets were located 45 degrees to the left of the sagittal plane and 15 cm away from the start position (Fig 1A). The rectangular target was rotated so that its major axis aligned with the center of the start position. For each trial, participants began from the start position and were instructed to “reach and stop inside the target.” The start position turned yellow after a short, randomized delay (250–1000 ms) to signal the beginning of the trial. Final hand position was defined as the participant’s hand location 100 ms after their hand velocity went below 0.045 cm/s. Participants would then receive feedback on task performance (see below). One second after stopping, the robot used a minimum jerk trajectory to return their hand to the start position.

During baseline (50 trials) and each block of washout trials (50 trials each), participants reached towards and attempted to stop within a white circular target (radius = 0.5 cm). For the first 40 baseline and the first 40 washout trials in a block, participants received error-based feedback in the form of a small cursor (radius = 0.25 cm) aligned with their final hand position. No feedback was given for the final 10 baseline and final 10 washout trials. For all experimental conditions, participants reached towards and attempted to stop within a long rectangular target that encourages sensorimotor exploration [3, 8]. Here the major target axis aligns with the task-redundant dimension, while the minor target axis corresponds to the task-relevant dimension (Fig 1A). Past work has shown that movement variability can vary between the movement extent and lateral direction [91, 92], which may impact exploratory behaviour. Thus, as in our previous work [3, 18], we designed the task such that all comparisons are made along the same movement direction (movement extent) corresponding to the target’s major axis. The major axis length of the target was 12 cm [3, 8, 18]. The minor axis length of the target was proportional to each participant’s lateral movement variability during the last 10 baseline trials [3, 18] (0.65*σ*, **Experiment 1**: 1.16 ± 0.41 cm; **Experiment 2**: 1.06 ± 0.31cm; **Experiment 3** Parkinson’s Disease: 1.07 ± 0.91 cm; **Experiment 3** Age-Matched Control: 0.96 ± 0.34 cm).

Participants were told that base compensation was $5.00 USD and they could earn an additional $5.00 USD performance bonus based on task performance. After completing the experiment, all participants received $10.00 USD irrespective of task performance.

### Experiment 1 design

In **Experiment 1** we addressed how reinforcement-based feedback and error-based feedback differentially influence exploration along task-redundant dimensions. Participants performed two conditions. In the first condition, participants received reinforcement feedback when they stopped within the target: 1) the target would expand, 2) they would hear a pleasant sound, and 3) they would earn a small monetary reward. In the second condition, participants received error feedback each time they stopped within or near the target: a small cursor (radius = 0.25 cm) would be placed at their final hand position. We expected to find greater exploration with reinforcement feedback compared to error feedback. For **Experiment 1**, participants performed 50 baseline trials, 200 experimental trials, 50 washout trials, and then another 200 experimental trials. Condition order was counterbalanced.

### Experiment 2 design

In **Experiment 2** we addressed how reinforcement-based feedback and error-based feedback interact to influence task-redundant exploration, while also replicating the results of **Experiment 1**. Participants performed three conditions (Fig 1C): isolated reinforcement feedback, isolated error feedback, or both simultaneous reinforcement & error feedback. As in **Experiment 1**, we expected to find greater exploration with isolated reinforcement feedback compared to isolated error feedback. Additionally, we expected to find exploration with both reinforcement & error feedback to be greater than isolated error feedback, but less than isolated reinforcement feedback. For **Experiment 2**, participants performed 50 baseline trials, 200 experimental trials, 50 washout trials, 200 experimental trials, 50 washout trials, and then another 200 experimental trials. Condition order was counterbalanced.

### Experiment 3 design

In **Experiment 3**, we recruited participants with Parkinson’s disease and neurotypical age-matched controls to gain mechanistic insight into the role of the basal ganglia and associated reinforcement (reward) pathways on sensorimotor exploration. As in **Experiment 2**, participants performed three conditions (Fig 1D): isolated reinforcement feedback, isolated error feedback, or reinforcement & error feedback simultaneously. We expected participants with Parkinson’s disease to display lower levels of exploration compared to age-matched controls in both the reinforcement condition and reinforcement & error condition. For **Experiment 3**, participants performed 50 baseline trials, 100 experimental trials, 50 washout trials, 100 experimental trials, 50 washout trials, and then another 100 experimental trials. Condition order was randomized across participants.

## Data analysis

We performed data analysis using custom Python 3.8.12 scripts. For all experiments, final hand position coordinates were projected onto a rotated cartesian coordinate system that was aligned with the major and minor axes of the rectangular targets [3, 18]. Thus the x-axis and y-axis of the rotated coordinate system aligned with the minor and major axis of the target respectively. The origin of this coordinate system was the center of the virtually displayed targets. In this study, we primarily focus on the major target axis as it aligns with the task-redundant dimension (Fig 1A). Focusing on the task-redundant dimension helps to mitigate the influence of cognitive processes, such as aiming strategies, while observing exploratory behaviour in response to failures along the task-relevant dimension.

### Quantifying exploration using Lag-1 autocorrelation

We [3, 18] and others [8] have used the lag-1 autocorrelation of final hand positions to quantify the amount of exploration along task dimensions. A larger lag-1 autocorrelation indicates greater exploration by using knowledge of movement variability to update movement aim [3]. Low lag-1 autocorrelations are associated with greater error corrections [8]. Unlike other metrics of exploration such as interquartile range and trial-by-trial movement variability, lag-1 autocorrelation captures the temporal structure of trial-by-trial data. The temporal structure of repeated movements can help to gain insight into if and how intended movements are updated on a trial-by-trial basis [3]. Lag-1 autocorrelation can take on a range of values, where a value of +1 would an update towards the previous reach every trial and a value of -1 would be over-correction on every trial (i.e. hand positions cross over the central axis every trial). Thus, a continuum of lag-1 autocorrelation values can occur due to a combination of movement updates toward the recent success and error-based corrections. In **Experiment 1** and **Experiment 2**, participants performed 200 reaches per condition, yielding 199 data points for the lag-1 autocorrelation analysis. In **Experiment 3**, participants performed 100 reaches per condition, yielding 99 data points for the lag-1 autocorrelation analysis. Utilizing a large number of trials in the lag-1 autocorrelation analysis mitigates the influence of the occasional large change in reach position.

We recently postulated that reinforcement feedback and error feedback may differentially affect task exploration [3], but we have yet to test this empirically. Additionally, it has been shown that Parkinson’s disease can decrease the magnitude of exploration following an unsuccessful action [6]. Thus, we also expected that Parkinson’s disease would disrupt the exploratory random walk behaviour of reinforcement-based exploration. As in our previous work [3, 18], we performed a lag-1 autocorrelation analysis separately along the major and minor axes of the rectangular target.

### Models of final hand position

#### Model 1 -task-relevant dimension: Correct to movement aim and target center; Task-redundant dimension: Correct to movement aim and target center

Reinforcement-based and error-based motor adaptation models have used various assumptions regarding how the sensorimotor system updates the intended movement aim following feedback. Here we have developed a general set of equations (Model 1) that models both reinforcement-based and error-based processes when reaching towards a target. Our general model also captures how these two processes act in concert with one another, since they are simultaneously used to update movement aim. We used this general model to make *a priori* predictions of 2D endpoint reach behaviour and resulting exploratory behaviour. For all models, we simulated final hand position along the task-relevant dimension (*X*^*t*^) and task-redundant dimension (*Y*^*t*^) as

$$\begin{array}{c} X_{t}=X_{t}^{aim}+\epsilon_{t}^{M,x}+\left(1-r_{t-1}\right)\epsilon_{t}^{E,x} \end{array}$$ (1A)

$$\begin{array}{c} Y_{t}=Y_{t}^{aim}+\epsilon_{t}^{M,y}+\left(1-r_{t-1}\right)\epsilon_{t}^{E,y} \end{array}$$ (1B)

$$\begin{array}{c} X_{t+1}^{aim}=X_{t}^{aim}+r_{t}\alpha^{x}\left[\left(1-r_{t-1}\right)\epsilon_{t}^{E,x}\right]-\beta^{aim,x}\left(X_{t}-X_{t}^{aim}\right)-\beta^{target,x}\left(X_{t}-T^{x}\right) \end{array}$$ (1C)

$$\begin{array}{c} Y_{t+1}^{aim}=Y_{t}^{aim}+r_{t}\alpha^{y}\left[\left(1-r_{t-1}\right)\epsilon_{t}^{E,y}\right]-\beta^{aim,y}\left(Y_{t}-Y_{t}^{aim}\right)-\beta^{target,y}\left(Y_{t}-T^{y}\right) \end{array}$$ (1D)

Final reach position on the current trial (*X*_*t*_, *Y*_*t*_) is equal to the intended movement aim ($X_{t}^{aim},Y_{t}^{aim}$) with additive Gaussian noise ($\epsilon_{t}^{i}∼N\left(0,\sigma_{i}^{2}\right)$). Superscripts represent the source of the variability: motor movement variability [56–58] (M) and exploratory movement variability (E). Note that exploratory movement variability is added only if the previous trial was unsuccessful [3–6] (*r*_*t*−1_ = 0). If the trial is successful (*r*_*t*_ = 1) and reinforcement feedback is given, the intended movement aim is updated proportionally (*α*) to exploratory movement variability [3]. On trials where visual feedback of the final hand position is given, movement aim is partially corrected towards the intended movement aim [54] (*β*^*aim*^) and partially corrected towards the center of the target [8, 10, 50–53] (*β*^*target*^).

We used Model 1 to make *a priori* predictions of both individual level and group level behaviour (Fig 2). During the reinforcement feedback condition, there are no acting error-based corrective processes (i.e. *β*^*i*^ = 0). During the error feedback condition, there are no acting reinforcement-based processes (i.e. *α*^*i*^ = 0). During the reinforcement & error feedback condition, there are acting reinforcement-based and error-based processes. Group level predictions are generated by simulating 500 individuals. Movement aim ($X_{t}^{aim},Y_{t}^{aim}$) was initialized to the target center (*T*^*x*^ = 0, *T*^*y*^ = 0). Model 1 has 10 free parameters.

As in our previous work [3], our goal was to find the simplest model that best explains our results across all three experiments. Models with large numbers of free parameters can be prone to overfitting. Thus, we systematically reduced the number of free parameters from the general model (Model 1). Each reduced model provides a different interpretation of the mechanism governing sensorimotor exploration and error correction. We used Akaike Information Criterion (AIC) and Bayesian Information Criterion (BIC) to capture how well a model fits the data given its number of free parameters. In addition to Model 1, we considered 8 additional models. For all models, the equations governing movement execution are the same. Consistent with our past work [3] each model uses the same reinforcement-based process of expanding movement variability after a miss, and updating movement aim after a success by using a portion of the movement variability. However, we systematically varied the model parameters responsible for error-based corrections. Each model tests a different mechanism by which the sensorimotor system may make corrections to movement aim when given visual error feedback. Model descriptions can be found in S1 Appendix.

### Experiment 1 & 2 model fitting

We used the same fitting procedure for each of our nine models [3]. Model fitting was performed using the Powell algorithm in the minimize function from the Scipy Python library.

For each model, we simulated 500 participants in each experiment to obtain a stable estimate of the mean lag-1 autocorrelation. We calculated the mean lag-1 autocorrelation along both the major and minor axes of the rectangular target. We defined the loss function as the difference between the average simulated and average participant lag-1 autocorrelations along the major and minor axes of the target. The optimizer minimized the sum of the loss across both **Experiments 1** and **2**.

The fitting procedure began with a “warm-start” where we minimized the model loss using a randomized initial parameter guess. We repeated this process 10,000 times. From these 10,000 initializations, we selected the set of parameters that resulted in the lowest final loss. These parameters were used as the initial guess of a bootstrapping procedure (10,000 iterations) to find the 95% confidence intervals of the posterior distribution for each free parameter. Participant lag-1 autocorrelations were randomly sampled with replacement for each iteration of the bootstrap procedure. The average lag-1 autocorrelation from this resampled group was used to determine the loss for that iteration. Both reinforcement-based (*α*) and error-based (*β*^*i*^) terms were bounded from 0 to 1. Variances used for movement variability terms were bounded based on the smallest and largest observed participant movement variability.

### Experiment 3 model fitting

In **Experiment 3**, we fit both the age-matched controls and participants with Parkinson’s disease simultaneously using the same set of movement variability terms, but a separate set of reinforcement-based (*α*) and error-based (*β*^*i*^) parameters for each group. This was done to capture the influence of Parkinson’s disease on the underlying mechanisms of sensorimotor exploration. Additionally, behavioural results suggest that movement variability following successful and unsuccessful trials is not significantly different between the group with Parkinson’s disease and age-matched control group (Fig 7). All other model fitting procedures were carried out as described in the section above.

### Best-fit model selection

We defined the median from the correspondent posterior distribution of each parameter as the best parameter set for each model. Using these median parameter values (Θ), we simulated each experiment and each condition with each model. We used a loss function (*L*_*i*_) that took the squared difference between the simulated (*ACF*^*Model*^) and average participant (*ACF*^*Data*^) lag-1 autocorrelation along the two target axes (j; major and minor) in each condition (k) for **Experiment 1** (Eq 10), **Experiment 2** (Eq 11), and both groups (l) in **Experiment 3** (Eq 12):

$$\begin{array}{c} L_{1}=\sum_{j=1}^{2}\sum_{k=1}^{2}{\left(ACF_{j,k}^{Data}-ACF_{j,k}^{Model}\left(\Theta \right)\right)}^{2} \end{array}$$ (10)

$$\begin{array}{c} L_{2}=\sum_{j=1}^{2}\sum_{k=1}^{3}{\left(ACF_{j,k}^{Data}-ACF_{j,k}^{Model}\left(\Theta \right)\right)}^{2} \end{array}$$ (11)

$$\begin{array}{c} L_{3}=\sum_{j=1}^{2}\sum_{k=1}^{3}\sum_{l=1}^{2}{\left(ACF_{j,k,l}^{Data}-ACF_{j,k,l}^{Model}\left(\Theta \right)\right)}^{2} \end{array}$$ (12)

$$\begin{array}{c} L_{total}=L_{1}+L_{2}+L_{3} \end{array}$$ (13)

By defining the loss function this way, a model that closely resembles the data across all experiments will result in a low final loss (*L*_*total*_). We used the loss from the best parameter set of each model for both the Bayesian Information Criteria (BIC) and Akaike Information Criteria (AIC) analyses. Using both analyses helps us select the simplest model that best represents the data. Bayesian Information Criteria (Eq 14) weights the number of free parameters (k) by the number of datapoints (n) used to calculate the loss (*L*_*total*_). Note that using a squared loss function is equivalent to calculating the likelihood function under the assumption of normality. We also considered Akaike Information Criteria (Eq 15) which uniformly weights free parameters (k) used in the model. Bayesian Information Criteria can be biased towards models with fewer parameters compared to Akaike Information Criteria. For both BIC and AIC analyses, a lower score indicates a better fit. We considered both analyses when selecting the best-fit model. We defined our best-fit model as the model that yielded the lowest BIC and AIC scores across all three experiments.

$$\begin{array}{c} BIC=k\;\text{ln}\left(n\right)+n\;\text{ln}\left(L_{total}\right) \end{array}$$ (14)

$$\begin{array}{c} AIC=2k+n\;\text{ln}\left(L_{total}\right) \end{array}$$ (15)

Parameter posterior probability distributions for the best-fit model can be found in Fig I in S1 Appendix. A limitation of our models is that they hold parameters constant when simulating multiple participants. That is, while they are stochastic and account for within participant variability, they do not consider between participant variability. Not accounting for between participant variability would to some degree likely influence plotted error bars in figures that contain simulations (e.g., Fig 4A and 4B). However through the bootstrapping procedure we are able to acquire confidence internals of model parameters that would account for the many possible sources of variance.

### Trial-by-trial movement variability

We [3, 18] and others [4–6, 62] have shown that movement variability is modulated by task outcome. Furthermore, Parkinson’s disease has been shown to reduce movement variability following an unsuccessful action compared to age-matched controls [6]. We defined movement variability as the standard deviation of the trial-by-trial change in reach position [3, 6, 18] (Δ*X*). Movement variability was calculated separately along the major and minor target axes. Movement variability within experimental conditions was also calculated separately for successful (target hit) and unsuccessful (target miss) reaches.

$$\begin{array}{c} \Delta X^{hit}=X_{t+1}-X_{t}^{hit} \end{array}$$ (16)

$$\begin{array}{c} \Delta X^{miss}=X_{t+1}-X_{t}^{miss} \end{array}$$ (17)

Here, X represents participant final hand position on a given trial (t). Superscripts represent whether the trial was successful (hit) or unsuccessful (miss).

### Statistical analysis

Non-parametric bootstrap hypothesis tests (1,000,000 iterations) were used for follow-up mean comparisons [3, 4, 18, 48, 93–97]. We used directional tests when testing theory-driven predictions, and non-directional tests otherwise. Spearman’s Rank correlation was used for all correlation measures. Common language effect size was computed for all mean comparisons. Statistical tests were considered significant at p < 0.05.

## Supporting information

S1 Appendix Fig A: Minor Axis Group Results for Experiment 1 & 2. Fig B: Parkinson’s and Age-Matched Control Minor Axis Results. Fig C: Wood et al., 2024 Model Fitting. Fig D: Minor Axis Task Outcome Movement Variability. Fig E: Major Axis Task Outcome Movement Variability. Fig F: Experiment 3 Group Comparison, Minor Axis Movement Variability Conditioned on Task Outcome. Fig G: Model Predictions for Major Axis Lag-1 Autocorrelation. Fig H: Model Predictions for Minor Axis Lag-1 Autocorrelation. Fig I: Best-fit Model Parameter Distribution. Fig J: Best-fit Model Parameter Distribution: Age-Matched Control. Fig K: Best-fit Model Parameter Distribution: Parkinson’s Disease. Fig L: Best-fit Model Parameter Distribution: Wood et al., 2024. Fig M: Absolute Change in Reach Position When Comparing Hits and Misses. Fig N: Absolute Change in Reach Aim When Comparing Between Conditions.
(PDF)
