# Supplementary material for: Roles and interplay of reinforcement-based and error-based processes during reaching and gait in neurotypical adults and individuals with Parkinson’s disease
Source: PLoS Comput Biol. 2024 Oct 14;20(10):e1012474. doi: 10.1371/journal.pcbi.1012474 (PMC11472932; doi:10.1371/journal.pcbi.1012474)
Supplement: S1 Appendix — Fig A: Minor Axis Group Results for Experiment 1 & 2. Fig B: Parkinson’s and Age-Matched Control Minor Axis Results. Fig C: Wood et al., 2024 Model Fitting. Fig D: Minor Axis Task Outcome Movement Variability. Fig E: Major Axis Task Outcome Movement Variability. Fig F: Experiment 3 Group Comparison, Minor Axis Movement Variability Conditioned on Task Outcome. Fig G: Model Predictions for Major Axis Lag-1 Autocorrelation. Fig H: Model Predictions for Minor Axis Lag-1 Autocorrelation. Fig I: Best-fit Model Parameter Distribution. Fig J: Best-fit Model Parameter Distribution: Age-Matched Control. Fig K: Best-fit Model Parameter Distribution: Parkinson’s Disease. Fig L: Best-fit Model Parameter Distribution: Wood et al., 2024. Fig M: Absolute Change in Reach Position When Comparing Hits and Misses. Fig N: Absolute Change in Reach Aim When Comparing Between Conditions. (PDF) [file pcbi.1012474.s001.pdf]

S1 Appendix

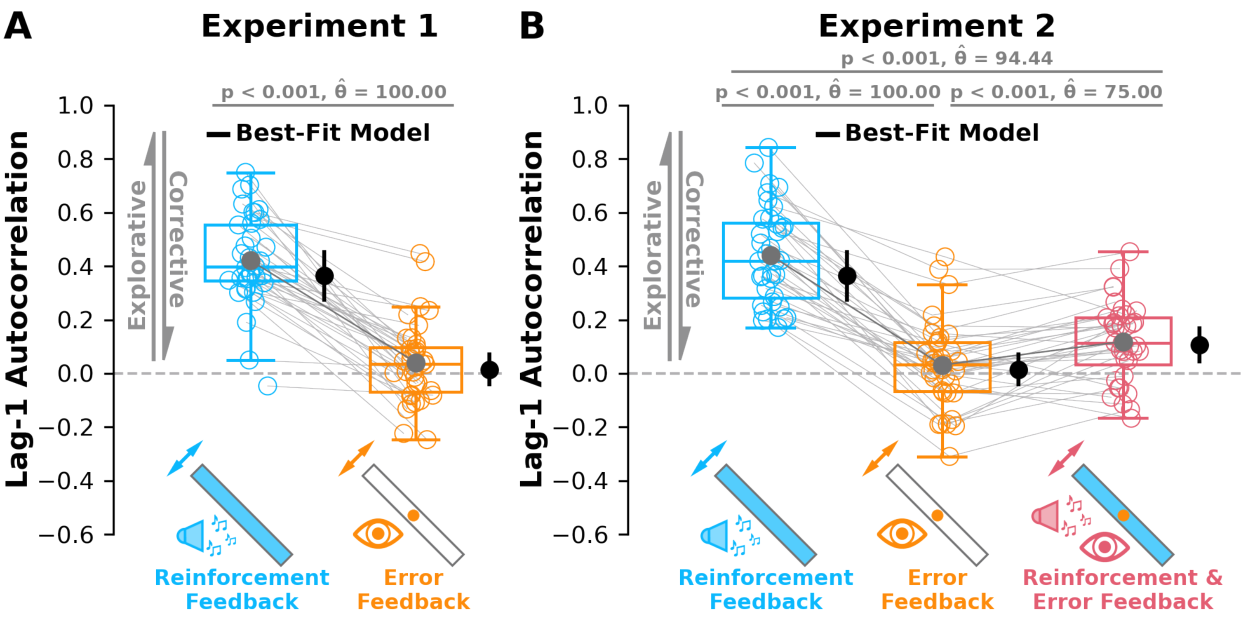

Fig A: Minor Axis Group Results for Experiment 1 & 2. A) Here we show lag-1 autocorrelation (y-axis) along the minor target axis for each participant in both conditions (x-axis) in **Experiment 1**. Aligning with model predictions (**Fig. 2D**), participants displayed significantly greater lag-1 autocorrelations in the reinforcement feedback condition (blue) compared to the error feedback condition (orange; $p < 0.001$). **B)** Lag-1 autocorrelation along the minor target axis (y-axis) for each condition (x-axis) in **Experiment 2**. Replicating the results of **Experiment 1**, participants in the reinforcement feedback condition (blue) again displayed greater lag-1 autocorrelations than the error feedback condition (orange; $p < 0.001$). Participants receiving both reinforcement & error feedback simultaneously displayed greater lag-1 autocorrelations compared to just error feedback ($p < 0.001$), but less than just reinforcement feedback ($p < 0.001$). These results suggest that reinforcement feedback boosts exploration while error feedback suppresses exploration. Moderate levels of exploration in the reinforcement & error feedback condition suggests that reinforcement-based and error-based processes interact. We performed a model comparison analysis to better understand the mechanism underlying sensorimotor exploration. Black solid circles and lines show the resulting mean and inner quartiles of the best-fit model simulations (Model 4) for both **A) Experiment 1** and **B) Experiment 2**. Box and whisker plots display the 25th, 50th, and 75th percentiles.

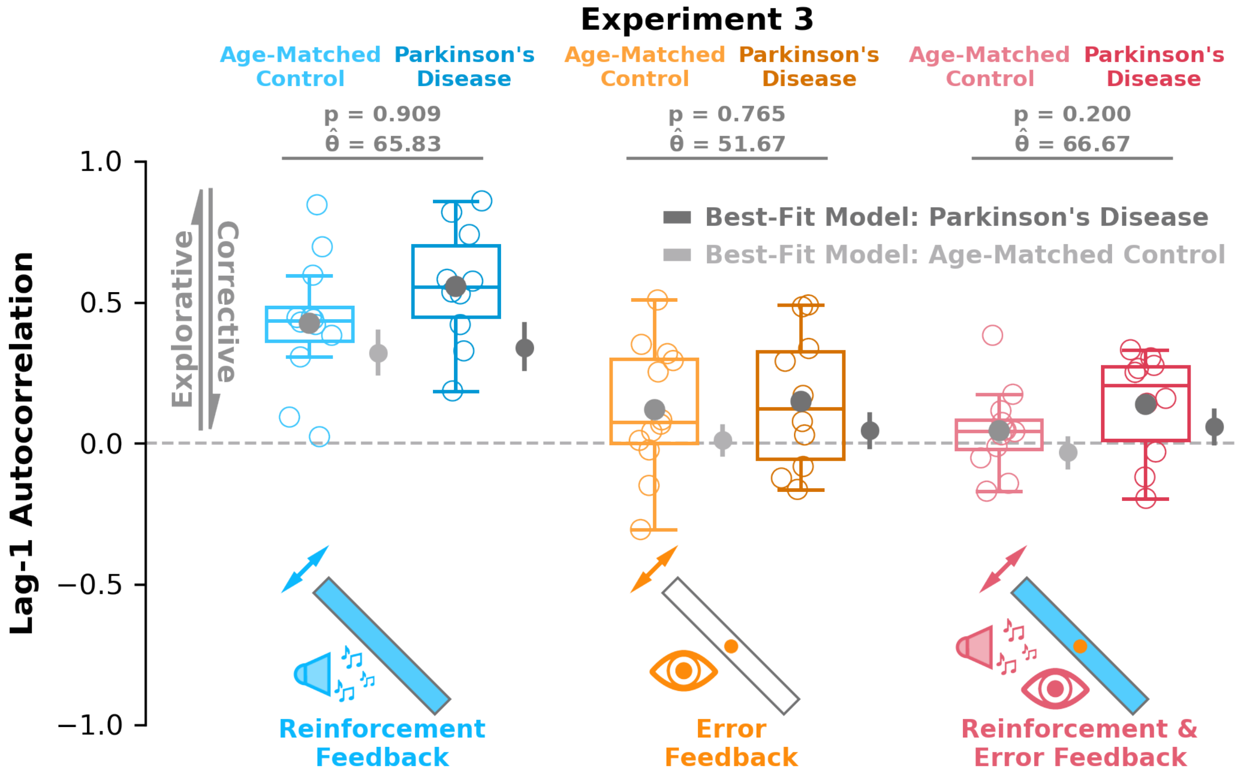

Fig B: Parkinson's and Age-Matched Control Minor Axis Results. A) Here we show lag-1 autocorrelation (y-axis) along the minor target axis for each participant in both conditions (x-axis) in **Experiment 1**. Aligning with model predictions (**Fig. 2D**), participants displayed significantly greater lag-1 autocorrelations in the reinforcement feedback condition (blue) compared to the error feedback condition (orange; $p < 0.001$). **B)** Lag-1 autocorrelation along the minor target axis (y-axis) for each condition (x-axis) in **Experiment 2**. Replicating the results of **Experiment 1**, participants in the reinforcement feedback condition (blue) again displayed greater lag-1 autocorrelations than the error feedback condition (orange; $p < 0.001$). Participants receiving both reinforcement & error feedback simultaneously displayed greater lag-1 autocorrelations compared to just error feedback ($p < 0.001$), but less than just reinforcement feedback ($p < 0.001$). These results suggest that reinforcement feedback boosts exploration while error feedback suppresses exploration. Moderate levels of exploration in the reinforcement & error feedback condition suggests that reinforcement-based and error-based processes interact. We performed a model comparison analysis to better understand the mechanism underlying sensorimotor exploration. Black solid circles and lines show the resulting mean and inner quartiles of the best-fit model simulations (Model 4) for both **A) Experiment 1** and **B) Experiment 2**. Box and whisker plots display the 25th, 50th, and 75th percentiles.

Wood et al., 2024 Re-Analysis

Wood et al., 2024 Methods

In the walking study by Wood et al. (2024), individuals learned to increase their left step length (the difference between the two heel positions in the anterior-posterior dimension at the time of left heel strike) based on one of two different types of visual feedback. The feedback was presented on a computer monitor placed in front of a dual belt treadmill with both belts moving at the same speed. After a no-feedback baseline phase, where participants in both groups were instructed to walk normally for 250 strides, they performed a 900-stride learning phase. Here, the left step length target was initially set at their baseline step length (50 strides), then the target gradually increased at a rate of 1% change from baseline every 10 strides until it reached 10%, where it held for the remainder of the learning phase (760 strides). The width of the target was always 4cm.

Two groups, a Reinforcement feedback group and an Error feedback group, learned this gait pattern using different feedback. The Error feedback group could see the step length target, represented as a pink horizontal target line, and a real time representation of their left step length relative to the target. The left step length was rendered as a blue bar, increasing vertically on the screen as their step length moved through the swing phase, then stopping once heel strike occurred. It stayed on the screen in that position until the next swing phase began. Before the learning phase, the Error feedback group was instructed to hit the target with each step. The Reinforcement feedback group was not given any visual feedback of step lengths or a visual target to provide magnitude or directional information. Instead, this group was provided only binary reward feedback, which took the form of a green check mark and money added to a total when their left step length landed within the invisible target zone. This group was instructed to gain as much money as possible and that the rewards were contingent on the length of the left step. Therefore, the Reinforcement feedback group had to find the correct step length, while the Error feedback group learned by correcting for visual errors in their left step length. While the learning phase was identical between Experiments 1 and 2 in the Wood study, here we focus on the data from Experiment 1.

Wood et al., 2024. Reinforcement-based processes boost exploration and error-based processes suppress exploration during gait

Exploratory behaviour has been seen across both reaching [1, 2, 3, 4, 5] and gait [6, 7, 8]. Recently, work by Wood and colleagues (2024) investigated how people learn a novel motor task with either reinforcement feedback or error feedback during gait. In their task, participants learned to adapt their left step length to stay within a target range that was longer than their baseline step length. In one group, participants received binary reinforcement feedback if their left step length fell within the target range. In another group, participants received error feedback in the form of a horizontal line representing the target and a vertically increasing bar representing their current left step length. The vertically increasing bar stopped at the end of each step to show participants how far they were from the target. In each group, the center of the target increased over subsequent strides until it reached a maximum step length. The center of the target remained stationary for the rest of the experiment. To minimize the influence of adaptation on our

metrics of exploration, we analyzed participant trial-by-trial left step lengths during the phase of the experiment after the target became stationary.

Figure B1 shows the lag-1 autocorrelation of each participant from the study by Wood and colleagues (2024). Aligning with our own results from both **Experiment 1** and **Experiment 2**, participants displayed significantly greater lag-1 autocorrelations in their gait when receiving reinforcement feedback compared to error feedback ($p < 0.001$; $\hat{\theta} = 100.0$). This suggests that reaching and gait may share a similar mechanism governing exploration when there is some degree of redundancy within the task. That is, reinforcement-based processes boost exploration while error-based processes suppress exploration in both reaching and gait.

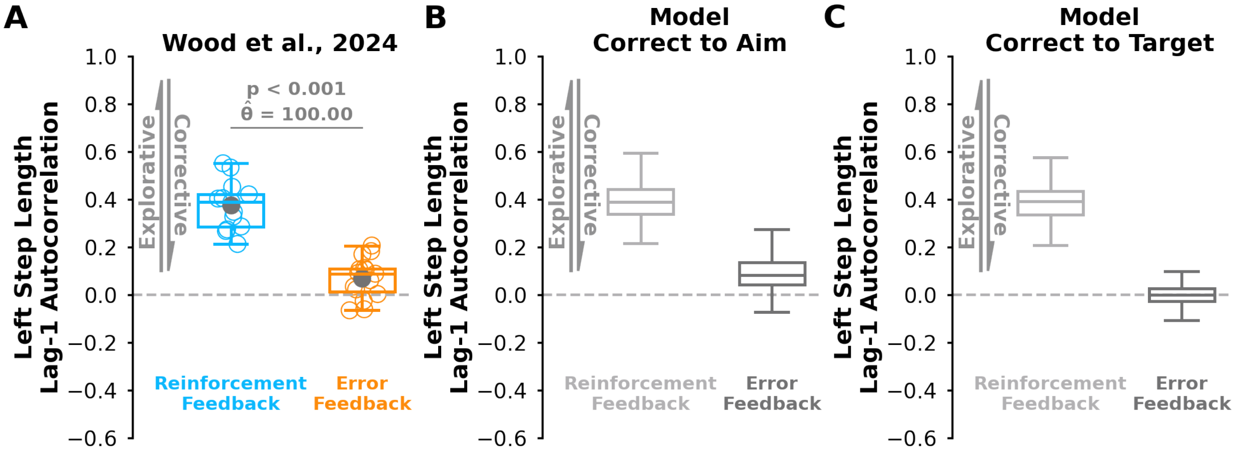

Fig C: Wood et al., 2024 Model Fitting. A) Here we show the lag-1 autocorrelation (y-axis) for the reinforcement (blue) and error (orange) groups from Wood et al. (2024) when the target remained fixed at +10% of their baseline step length. The reinforcement group displayed significantly greater lag-1 autocorrelation compared to the error group ($p < 0.001$). Along the task-redundant dimension, our best-fit model (Model 4) makes error corrections to the intended movement aim. Here, we tested both an error signal relative to the target center as well as an error signal relative to the previously intended movement aim in gait. We fit the model separately using either of these two correction mechanisms: **B)** correcting to the movement aim and **C)** correcting to the target center. The modelling results suggest that corrective actions were made in reference to the movement aim in the error feedback group, rather than the center of the target, matching the task-redundant dimension of our best-fit model (Model 4) in reaching.

Exploratory random walk behaviour has previously been found in gait patterns [6, 7]. Here we show for the first time that exploratory random walk behaviour in gait is differentially influenced by reinforcement-based and error-based processes. Additionally, the best-fit model (Model 4) did well to capture both our findings in reaching and the Wood and colleagues (2024) findings in gait, further suggesting similar reinforcement-based and error-based mechanisms for both reaching and gait. However, there is notably less exploratory random walk behaviour in the Wood (2024) gait dataset compared to our reaching experiments, which may reflect additional stability costs during gait. That is, it is possible that additional stability costs made left step length more task-relevant in the Wood (2024) study. Another possibility is that in their task, there was a lesser degree of task redundancy compared to our reaching task. In other words, there was a smaller range of redundant solutions during gait compared to our reaching experiment. Thus,

despite displaying similar trends, either greater stability costs or less task redundancy may have contributed lower levels of exploratory random walk behaviour during gait compared to reaching.

Other metrics of exploration, such as greater exploratory movement variability following an unsuccessful action, have been shown in both prior reaching studies [1, 2, 4, 9, 10] as well as the gait study by Wood and colleagues (2024). Taken together, our study and Wood (2024) study support the idea that reaching and gait share similar exploratory mechanisms. A shared exploratory mechanism between reaching and gait could suggest that rehabilitation strategies may apply across reaching and gait. Indeed, past work has show that challenging stability related metrics, such as balance, can improve movement generalization across both reaching and gait [11, 12]. In **Experiment 2**, we found that reinforcement-based and error-based processes interact to influence exploratory random walk behaviour. It would be interesting for future work to investigate if this interaction is also present in gait. Additionally, in **Experiment 3** we found that Parkinson's disease impairs reinforcement-based exploratory behaviour in reaching. It may be useful for future work to investigate if this impaired exploratory mechanism due to Parkinson's disease persists in gait.

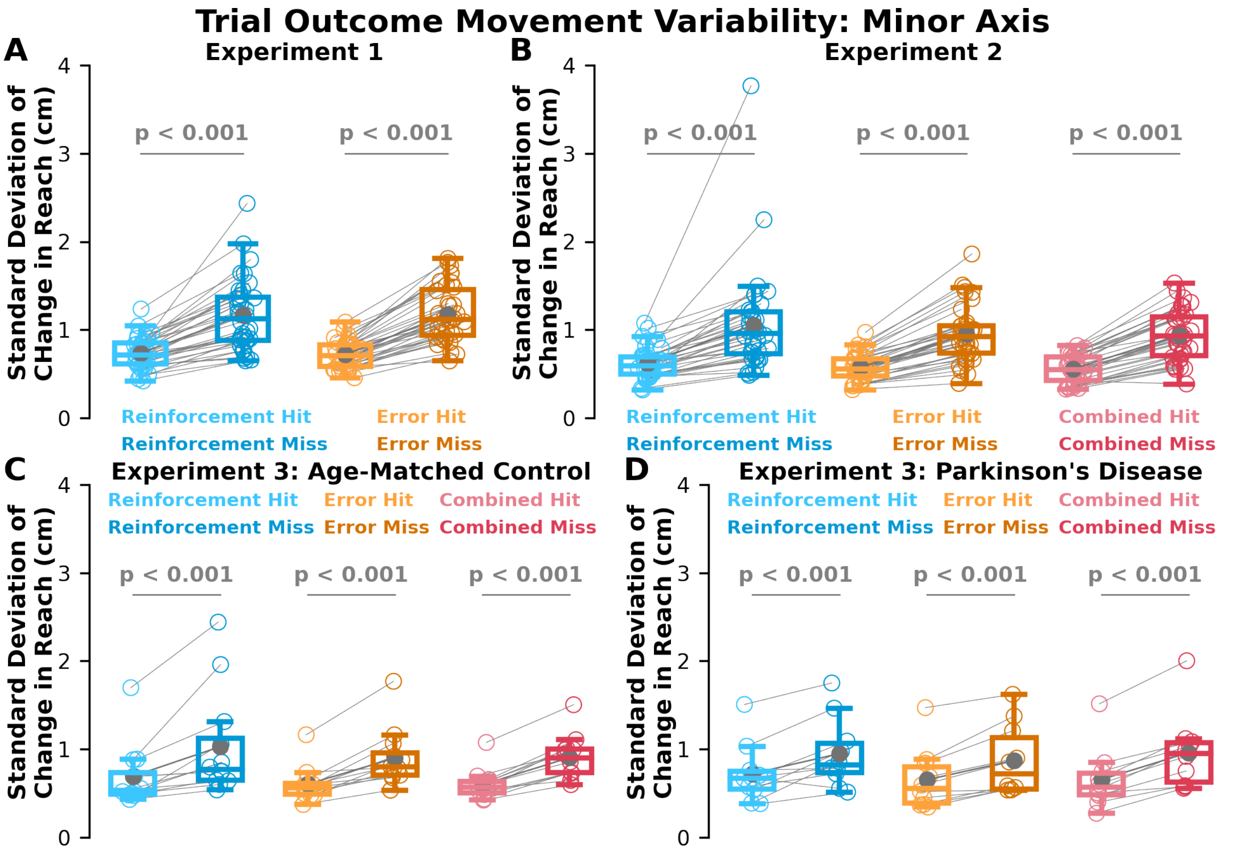

Fig D: Minor Axis Task Outcome Movement Variability. **A-D)** We calculated movement variability as the standard deviation of the trial-by-trial change in reach position along the minor target axis (y-axis) for each condition (x-axis). We calculated movement variability separately following successful trials (Hit, light colours) and unsuccessful trials (Miss, dark colours). **A)** Here we show movement variability following task outcome for the reinforcement feedback (blue) and error feedback (orange) conditions of **Experiment 1**. In both conditions, participants displayed significantly greater trial-by-trial movement variability following target misses compared to target hits ($p < 0.001$ for both conditions). **B)** In **Experiment 2**, we analyzed movement variability following target hits and misses for the reinforcement feedback, error feedback, and reinforcement & error feedback (combined, pink) condition. Across all three conditions, participants displayed significantly greater movement variability following a target miss compared to a target hit ($p < 0.001$ for all comparisons). In **Experiment 3**, we analyzed movement variability following a target hit or target miss for both **C)** the age-matched control group and **D)** the group with Parkinson's disease. Across both groups, participants displayed significantly greater movement variability following a target miss compared to a target hit ($p < 0.001$ for all comparisons). Box and whisker plots display the 25th, 50th, and 75th percentiles

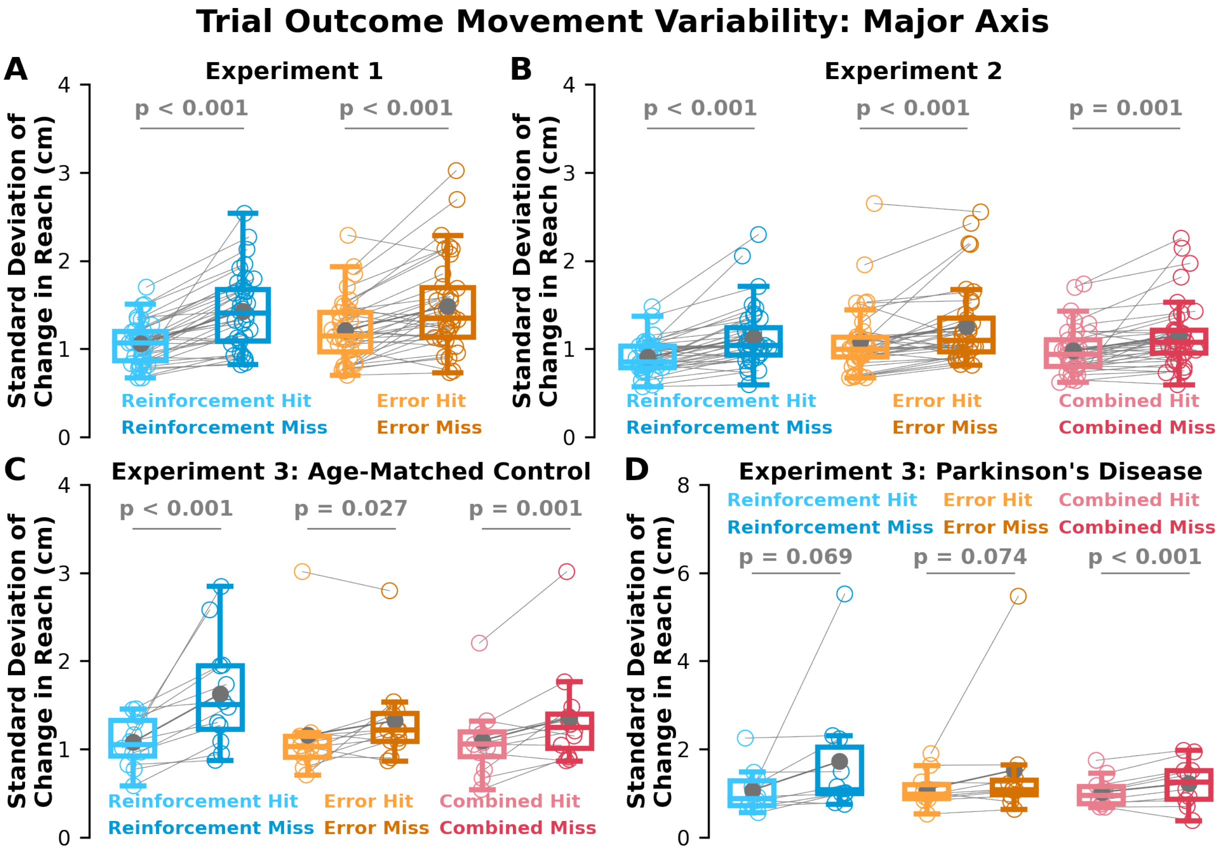

Fig E: Major Axis Task Outcome Movement Variability. **A-D)** We calculated movement variability as the standard deviation of the trial-by-trial change in reach position along the major target axis (y-axis) for each condition (x-axis). We calculated movement variability separately following successful trials (Hit, light colours) and unsuccessful trials (Miss, dark colours). **A)** Here we show movement variability following task outcome for the reinforcement feedback (blue) and error feedback (orange) conditions of **Experiment 1**. In both conditions, participants displayed significantly greater trial-by-trial movement variability following target misses compared to target hits ($p < 0.001$ for both conditions). **B)** In **Experiment 2**, we analyzed movement variability following target hits and misses for the reinforcement feedback, error feedback, and reinforcement & error feedback (combined, pink) condition. Across all three conditions, participants displayed significantly greater movement variability following a target miss compared to a target hit ($p < 0.001$ for all comparisons). **C)** In the age-matched control group of **Experiment 3**, participants displayed significantly greater movement variability following a target miss compared to a target hit across all three conditions ($p \leq 0.027$ for all comparisons). **D)** In the group with Parkinson's disease during **Experiment 3**, participants displayed significantly greater movement variability following a target miss compared to a target hit in the reinforcement & error (combined, pink) feedback condition ($p < 0.001$). Box and whisker plots display the 25th, 50th, and 75th percentiles

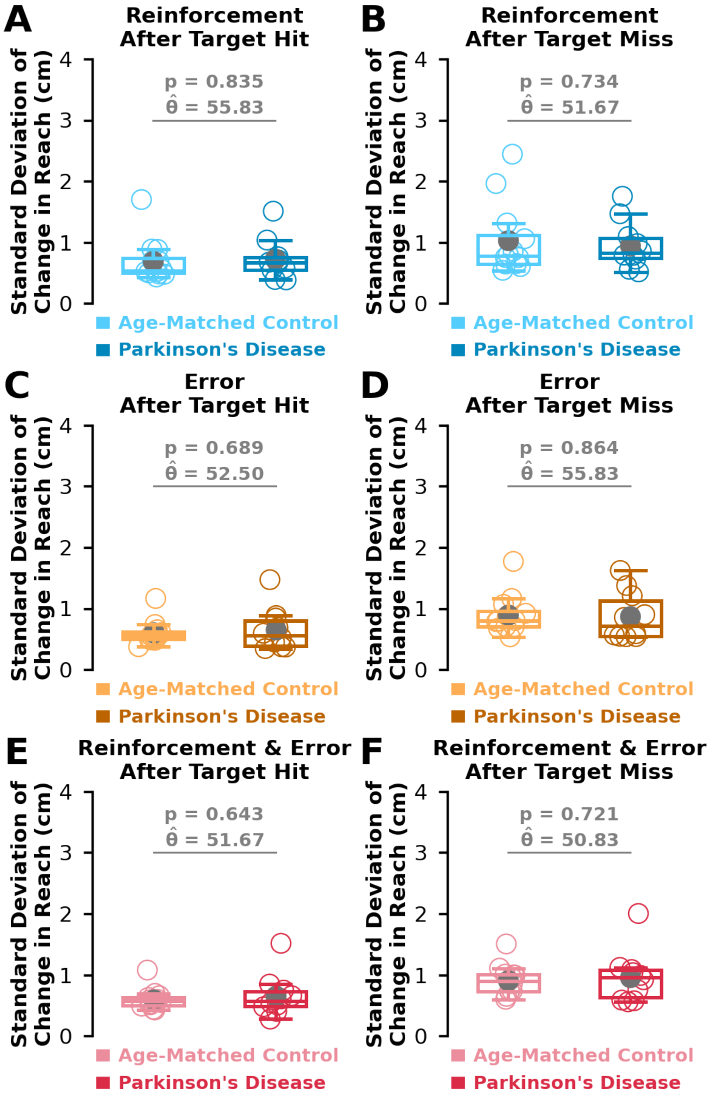

Fig F: Experiment 3 Group Comparison, Minor Axis Movement Variability Conditioned on Task Outcome. A-F) We calculated trial-by-trial movement variability as the standard deviation of change in reach (y-axis) following target hits (left column) and target misses (right column) for both the age-matched control group (light colors) and Parkinson's group (dark colors). Again, we did not find a significant difference between the age-matched control group and the Parkinson's group for movement variability following a target hit or a target miss in any of the three conditions. These results suggest that Parkinson's disease did not reduce trial-by-trial movement variability as suggested by prior literature [10]. Box and whisker plots display the 25th, 50th, and 75th percentiles. Hollow circles represent individual data. Solid circles represent group means.

All Model Simulations

Model 1 (General Model) - Task-relevant Dimension: Correct to Movement Aim and Target Center; Task-redundant Dimension: Correct to Movement Aim and Target Center.

$$X_t = X_t^{aim} + \epsilon_t^{M,x} + (1 - r_{t-1})\epsilon_t^{E,x} \tag{1A}$$
$$Y_t = Y_t^{aim} + \epsilon_t^{M,y} + (1 - r_{t-1})\epsilon_t^{E,y} \tag{1B}$$
$$X_{t+1}^{aim} = X_t^{aim} + r_t\alpha^x[(1 - r_{t-1})\epsilon_t^{E,x}] - \beta^{aim,x}(X_t - X_t^{aim}) - \beta^{target,x}(X_t - T^x) \tag{1C}$$
$$Y_{t+1}^{aim} = Y_t^{aim} + r_t\alpha^y[(1 - r_{t-1})\epsilon_t^{E,y}] - \beta^{aim,y}(Y_t - Y_t^{aim}) - \beta^{target,y}(Y_t - T^y) \tag{1D}$$

Model 2 (remove $\beta^{target,x}$, $\beta^{target,y}$) - Task-relevant Dimension: Correct to Movement Aim; Task-redundant Dimension: Correct to Movement Aim.

Our general model (Model 1) considers an error signal relative to the center of the target $(X_t - T^x)$ as well as an error signal relative to the intended movement aim $(X_t - X_t^{aim})$. Here, we consider a model that only makes movement aim corrections using an error signal relative to the previously intended movement aim. Model 2 has 8 free parameters.

$$X_t = X_t^{aim} + \epsilon_t^{M,x} + (1 - r_{t-1})\epsilon_t^{E,x} \tag{2A}$$
$$Y_t = Y_t^{aim} + \epsilon_t^{M,y} + (1 - r_{t-1})\epsilon_t^{E,y} \tag{2B}$$
$$X_{t+1}^{aim} = X_t^{aim} + r_t\alpha^x[(1 - r_{t-1})\epsilon_t^{E,x}] - \beta^{aim,x}(X_t - X_t^{aim}) \tag{2C}$$
$$Y_{t+1}^{aim} = Y_t^{aim} + r_t\alpha^y[(1 - r_{t-1})\epsilon_t^{E,y}] - \beta^{aim,y}(Y_t - Y_t^{aim}) \tag{2D}$$

Model 3 (remove $\beta^{aim,x}$, $\beta^{aim,y}$)- Task-relevant Dimension: Correct to Target Center; Task-redundant Dimension: Correct to Target Center

Our general model (Model 1) considers an error signal relative to the center of the target $(X_t - T^x)$, as well as an error signal relative to the intended movement aim $(X_t - X_t^{aim})$. Here we consider a model that only corrects its movement aim using an error signal relative to the center of the target. Model 3 has 8 free parameters.

$$X_t = X_t^{aim} + \epsilon_t^{M,x} + (1 - r_{t-1})\epsilon_t^{E,x} \tag{3A}$$
$$Y_t = Y_t^{aim} + \epsilon_t^{M,y} + (1 - r_{t-1})\epsilon_t^{E,y} \tag{3B}$$
$$X_{t+1}^{aim} = X_t^{aim} + r_t\alpha^x[(1 - r_{t-1})\epsilon_t^{E,x}] - \beta^{target,x}(X_t - T^x) \tag{3C}$$
$$Y_{t+1}^{aim} = Y_t^{aim} + r_t\alpha^y[(1 - r_{t-1})\epsilon_t^{E,y}] - \beta^{target,y}(Y_t - T^y) \tag{3D}$$

Model 4 (remove $\beta^{target,y}$) - Task-relevant Dimension: Correct to Movement Aim and Target Center; Task-redundant Dimension: Correct to Movement Aim

It is possible that different error signals are prioritized based on the relevance/redundancy of the movement dimension. Thus we consider a model that utilizes a different combination of error signals along its movement dimensions. Along the task-relevant dimension, Model 4 utilizes both an error signal relative to the target center and an error signal relative to the previously intended movement aim. Along the task-redundant dimension,

Model 4 only utilizes an error signal relative to the previously intended movement aim. Model 4 uses 9 free parameters.

$$X_t = X_t^{aim} + \epsilon_t^{M,x} + (1 - r_{t-1})\epsilon_t^{E,x} \tag{4A}$$

$$Y_t = Y_t^{aim} + \epsilon_t^{M,y} + (1 - r_{t-1})\epsilon_t^{E,y} \tag{4B}$$

$$X_{t+1}^{aim} = X_t^{aim} + r_t\alpha^x[(1 - r_{t-1})\epsilon_t^{E,x}] - \beta^{aim,x}(X_t - X_t^{aim}) - \beta^{target,x}(X_t - T^x) \tag{4C}$$

$$Y_{t+1}^{aim} = Y_t^{aim} + r_t\alpha^y[(1 - r_{t-1})\epsilon_t^{E,y}] - \beta^{aim,y}(Y_t - Y_t^{aim}) \tag{4D}$$

Model 5 (remove $\beta^{aim,y}$) - Task-relevant Dimension: Correct to Movement Aim and Target Center; Task-redundant Dimension: Correct to Target Center.

Along the task-relevant dimension, Model 5 utilizes both an error signal relative to the target center and an error signal relative to the previously intended movement aim. Along the task-redundant dimension, Model 5 only utilizes an error signal relative to the target center. Model 5 uses 9 free parameters.

$$X_t = X_t^{aim} + \epsilon_t^{M,x} + (1 - r_{t-1})\epsilon_t^{E,x} \tag{5A}$$

$$Y_t = Y_t^{aim} + \epsilon_t^{M,y} + (1 - r_{t-1})\epsilon_t^{E,y} \tag{5B}$$

$$X_{t+1}^{aim} = X_t^{aim} + r_t\alpha^x[(1 - r_{t-1})\epsilon_t^{E,x}] - \beta^{aim,x}(X_t - X_t^{aim}) - \beta^{target,x}(X_t - T^x) \tag{5C}$$

$$Y_{t+1}^{aim} = Y_t^{aim} + r_t\alpha^y[(1 - r_{t-1})\epsilon_t^{E,y}] - \beta^{target,y}(Y_t - T^y) \tag{5D}$$

Model 6 (remove $\beta^{aim,x}$) - Task-relevant Dimension: Correct to Target Center; Task-redundant Dimension: Correct to Movement Aim and Target Center

Along the task-relevant dimension, Model 6 only utilizes an error signal relative to the target center. Along the task-redundant dimension, Model 6 only utilizes both an error signal relative to the target center and an error signal relative to the previously intended movement aim. Model 6 uses 9 free parameters.

$$X_t = X_t^{aim} + \epsilon_t^{M,x} + (1 - r_{t-1})\epsilon_t^{E,x} \tag{6A}$$

$$Y_t = Y_t^{aim} + \epsilon_t^{M,y} + (1 - r_{t-1})\epsilon_t^{E,y} \tag{6B}$$

$$X_{t+1}^{aim} = X_t^{aim} + r_t\alpha^x[(1 - r_{t-1})\epsilon_t^{E,x}] - \beta^{target,x}(X_t - T^x) \tag{6C}$$

$$Y_{t+1}^{aim} = Y_t^{aim} + r_t\alpha^y[(1 - r_{t-1})\epsilon_t^{E,y}] - \beta^{aim,y}(Y_t - Y_t^{aim}) - \beta^{target,y}(Y_t - T^y) \tag{6D}$$

Model 7 (remove $\beta^{target,x}$) - Task-relevant Dimension: Correct to Movement Aim; Task-redundant Dimension: Correct to Movement Aim and Target Center

Along the task-relevant dimension, Model 7 only utilizes an error signal relative to the previously intended movement aim. Along the task-redundant dimension, Model 7 utilizes both an error signal relative to the target center and an error signal relative to the previously intended movement aim. Model 7 uses 9 free parameters.

$$X_t = X_t^{aim} + \epsilon_t^{M,x} + (1 - r_{t-1})\epsilon_t^{E,x} \tag{7A}$$

$$Y_t = Y_t^{aim} + \epsilon_t^{M,y} + (1 - r_{t-1})\epsilon_t^{E,y} \tag{7B}$$

$$X_{t+1}^{aim} = X_t^{aim} + r_t\alpha^x[(1 - r_{t-1})\epsilon_t^{E,x}] - \beta^{aim,x}(X_t - X_t^{aim}) \tag{7C}$$

$$Y_{t+1}^{aim} = Y_t^{aim} + r_t\alpha^y[(1 - r_{t-1})\epsilon_t^{E,y}] - \beta^{aim,y}(Y_t - Y_t^{aim}) - \beta^{target,y}(Y_t - T^y) \tag{7D}$$

Model 8 (remove $\beta^{target,x}$, $\beta^{aim,y}$) - Task-relevant Dimension: Correct to Movement Aim; Task-redundant Dimension: Correct to Target Center

Along the task-relevant dimension, Model 8 only utilizes an error signal relative to the previously intended movement aim. Along the task-redundant dimension, Model 8 only utilizes an error signal relative to the target center. Model 8 uses 8 free parameters.

$$X_t = X_t^{aim} + \epsilon_t^{M,x} + (1 - r_{t-1})\epsilon_t^{E,x} \tag{8A}$$

$$Y_t = Y_t^{aim} + \epsilon_t^{M,y} + (1 - r_{t-1})\epsilon_t^{E,y} \tag{8B}$$

$$X_{t+1}^{aim} = X_t^{aim} + r_t\alpha^x[(1 - r_{t-1})\epsilon_t^{E,x}] - \beta^{aim,x}(X_t - X_t^{aim}) \tag{8C}$$

$$Y_{t+1}^{aim} = Y_t^{aim} + r_t\alpha^y[(1 - r_{t-1})\epsilon_t^{E,y}] - \beta^{target,y}(Y_t - T^y) \tag{8D}$$

Model 9 (remove $\beta^{aim,x}$, $\beta^{target,y}$) - Task-relevant Dimension: Correct to Target Center; Task-redundant Dimension: Correct to Movement Aim

Along the task-relevant dimension, Model 9 only utilizes an error signal relative to the target center. Along the task-redundant dimension, Model 9 only utilizes an error signal relative to the previously intended movement aim. Model 9 uses 8 free parameters.

$$X_t = X_t^{aim} + \epsilon_t^{M,x} + (1 - r_{t-1})\epsilon_t^{E,x} \tag{9A}$$

$$Y_t = Y_t^{aim} + \epsilon_t^{M,y} + (1 - r_{t-1})\epsilon_t^{E,y} \tag{9B}$$

$$X_{t+1}^{aim} = X_t^{aim} + r_t\alpha^x[(1 - r_{t-1})\epsilon_t^{E,x}] - \beta^{target,x}(X_t - T^x) \tag{9C}$$

$$Y_{t+1}^{aim} = Y_t^{aim} + r_t\alpha^y[(1 - r_{t-1})\epsilon_t^{E,y}] - \beta^{aim,y}(Y_t - Y_t^{aim}) \tag{9D}$$

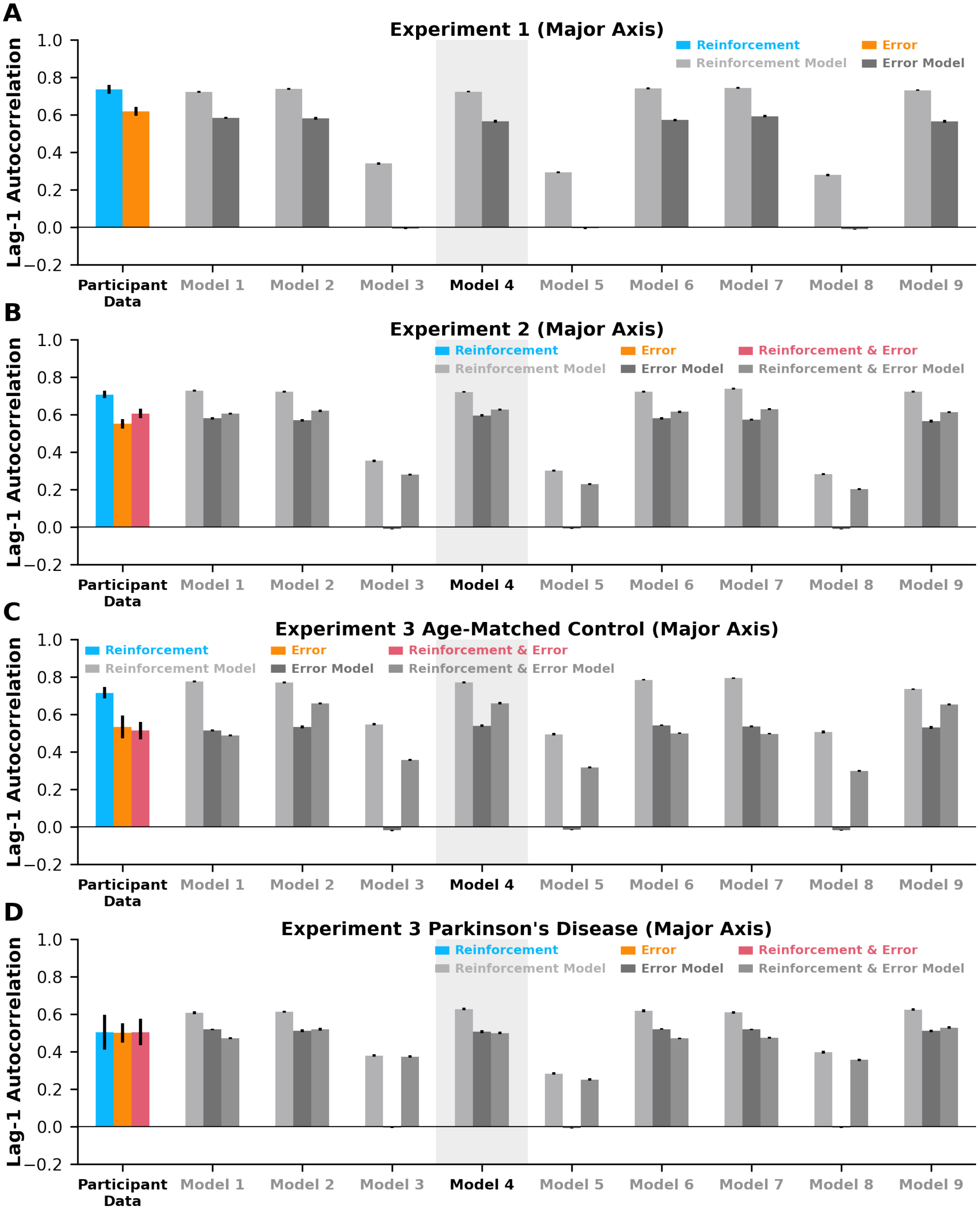

Fig G: Model Predictions for Major Axis Lag-1 Autocorrelation. We simulated 500 participants using each model's best fit parameters. Here we show the lag-1 autocorrelation (y-axis) from the resulting simulations for each model (x-axis). **A)** Participant lag-1 autocorrelations from **Experiment 1** along the major target axis for the reinforcement (blue) and error (orange) conditions. Simulations for the reinforcement (light grey) and error (dark grey) conditions are shown for each model. **B)** Participant lag-1 autocorre-

lations from **Experiment 2** along the major target axis for the reinforcement (blue), error (orange), and reinforcement & error (pink) conditions. Simulations for the reinforcement (light grey), error (dark grey), and reinforcement & error (grey) conditions are shown for each model. **C)** Participant lag-1 autocorrelations from age-matched control group along the major target axis in the reinforcement (blue), error (orange), and reinforcement & error (pink) conditions in **Experiment 3**. Simulations of the age-matched control group for the reinforcement (light grey), error (dark grey), and reinforcement & error (grey) conditions are shown for each model. **D)** Participant lag-1 autocorrelations from the Parkinson's disease group along the major target axis group in the reinforcement (blue), error (orange), and reinforcement & error (pink) conditions in **Experiment 3**. Simulations of the age-matched control group for the reinforcement (light grey), error (dark grey), and reinforcement & error (grey) conditions are shown for each model.

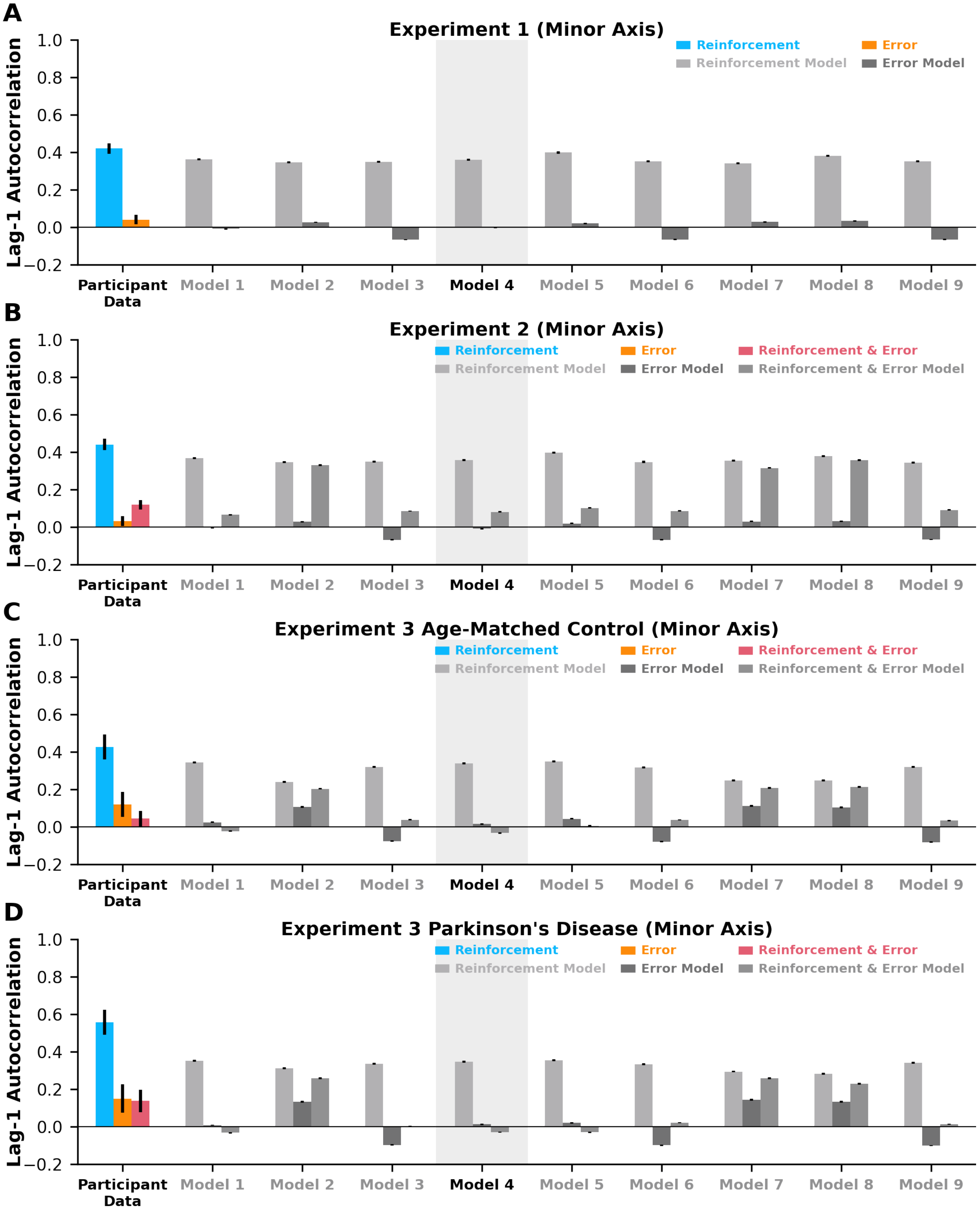

Fig H: Model Predictions for Minor Axis Lag-1 Autocorrelation. We simulated 500 participants using each model's best fit parameters. Here we show the lag-1 autocorrelation (y-axis) from the resulting simulations for each model (x-axis). **A)** Participant lag-1 autocorrelations from **Experiment 1** along the minor target axis for the reinforcement (blue) and error (orange) conditions. Simulations for the reinforcement (light grey) and error (dark grey) conditions are shown for each model. **B)** Participant lag-1 autocorre-

lations from **Experiment 2** along the minor target axis for the reinforcement (blue), error (orange), and reinforcement & error (pink) conditions. Simulations for the reinforcement (light grey), error (dark grey), and reinforcement & error (grey) conditions are shown for each model. **C)** Participant lag-1 autocorrelations from the age-matched control group along the minor target axis in the reinforcement (blue), error (orange), and reinforcement & error (pink) conditions in **Experiment 3**. Simulations of the age-matched control group for the reinforcement (light grey), error (dark grey), and reinforcement & error (grey) conditions are shown for each model. **D)** Participant lag-1 autocorrelations from **Experiment 3** Parkinson's disease group along the minor target axis in the reinforcement (blue), error (orange), and reinforcement & error (pink) conditions in **Experiment 3**. Simulations of the age-matched control group for the reinforcement (light grey), error (dark grey), and reinforcement & error (grey) conditions are shown for each model.

Supplementary E: Best-fit Model (Model 4) Parameter Distributions

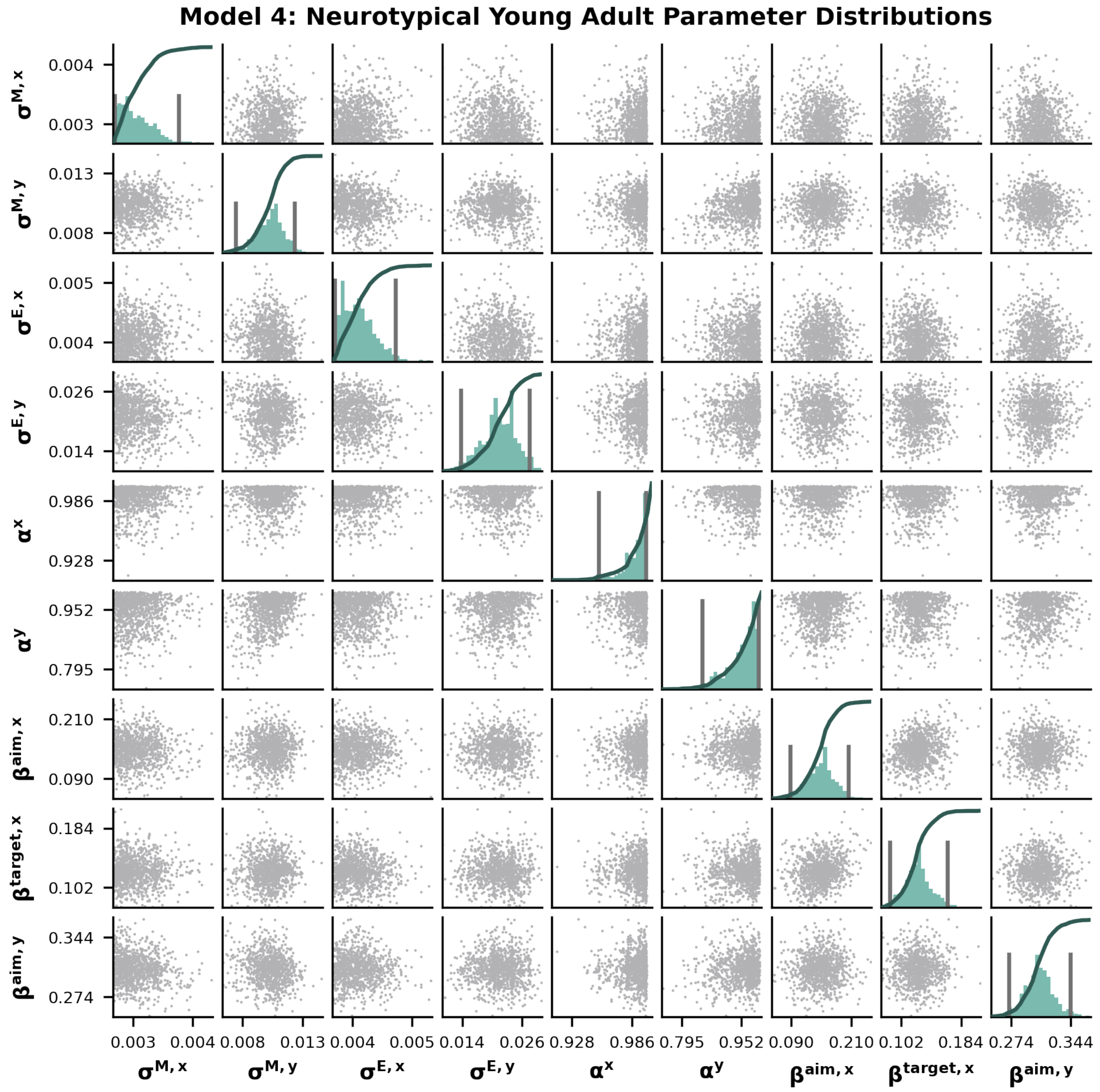

Fig I: Best-fit Model Parameter Distribution. A) Model parameters for the best-fit model (Model 4). Bootstrapped (10,000 samples) marginal probability distributions of each parameter value are shown along the diagonal (teal). 95% confidence intervals are indicated with dark grey lines. Thick dark teal lines correspond to the cumulative distribution. We used the median value for $\sigma^{M,x}$ (0.35 cm), $\sigma^{M,y}$ (1.04 cm), $\sigma^{E,x}$ (0.36 cm), $\sigma^{E,y}$ (2.16 cm), α^x (0.99), α^y (0.96), $\beta^{aim,x}$ (0.15), $\beta^{target,x}$ (0.12), $\beta^{aim,y}$ (0.31) to run simulations of **Experiment 1** and **Experiment 2** with the best-fit model. Off-diagonal plots show joint probability distributions for each pair of parameters.

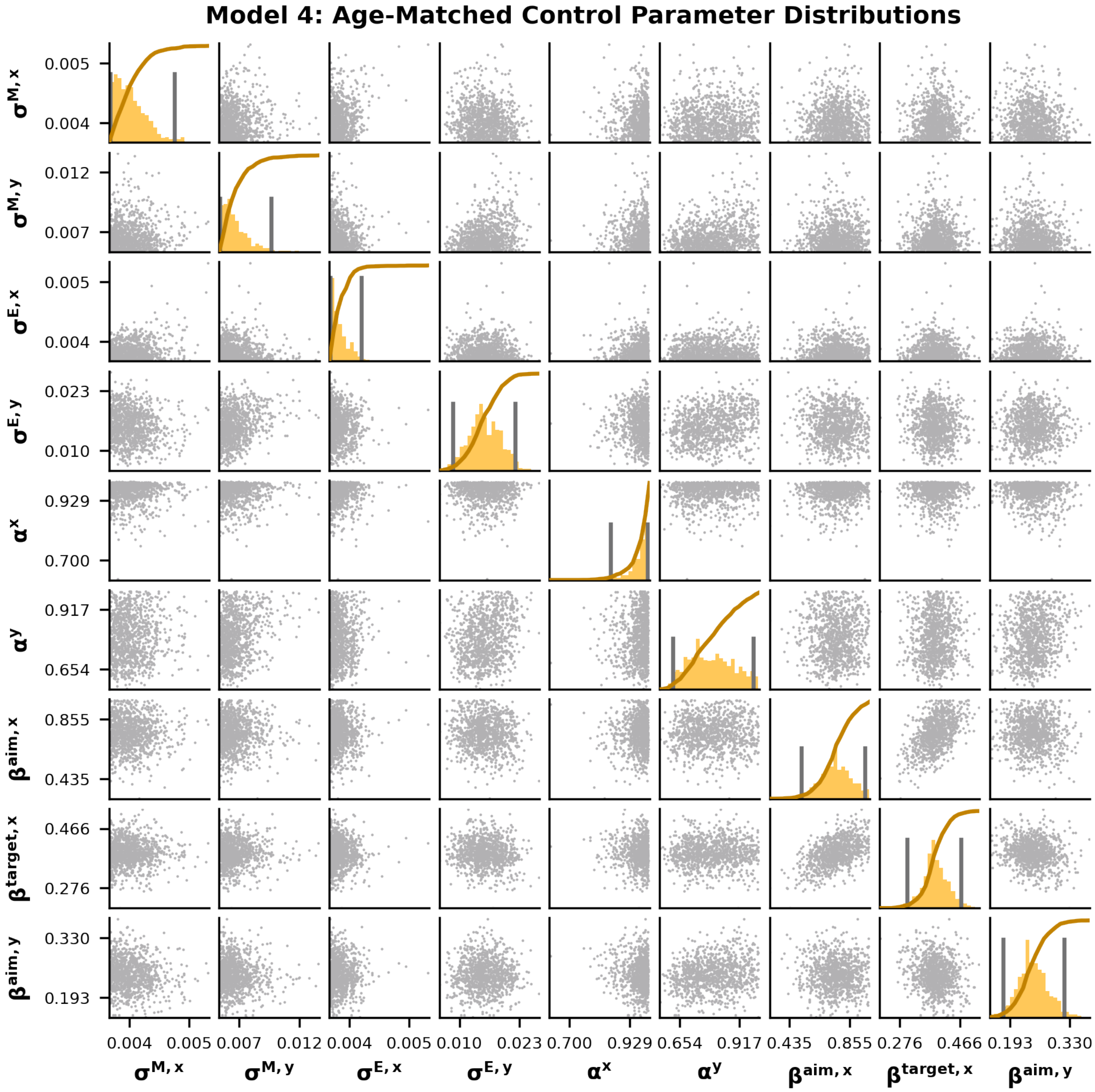

Fig J: Best-fit Model Parameter Distribution: Age-Matched Control. A) Model parameters for the best-fit model (Model 4). Bootstrapped (10,000 samples) marginal probability distributions of each parameter value are shown along the diagonal (yellow). 95% confidence intervals are indicated with dark grey lines. Thick dark yellow lines correspond to the cumulative distribution. We used the median value for $\sigma^{M,x}$ (0.41 cm), $\sigma^{M,y}$ (0.63 cm), $\sigma^{E,x}$ (0.38 cm), $\sigma^{E,y}$ (1.51 cm), α^{x} (0.98), α^{y} (0.79), $\beta^{aim,x}$ (0.75), $\beta^{target,x}$ (0.39), $\beta^{aim,y}$ (0.24) to run simulations of the age-matched control group in **Experiment 3** with the best-fit model. Off-diagonal plots show joint probability distributions for each pair of parameters. As a reminder, the Parkinson's disease group and age-matched control group share the same movement variability terms ($\sigma^{M,x}$, $\sigma^{M,y}$, $\sigma^{E,x}$, $\sigma^{E,y}$).

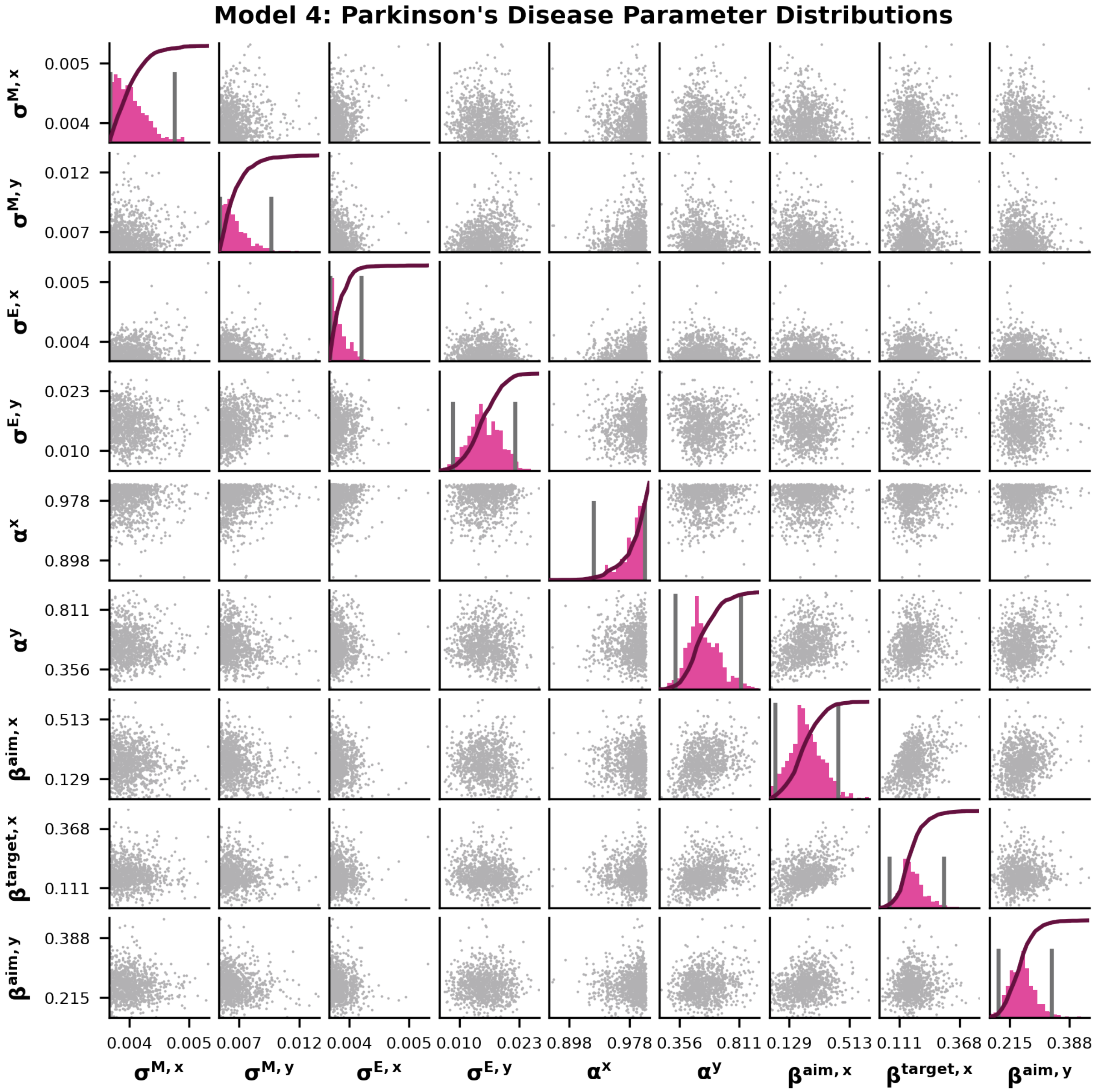

Fig K: Best-fit Model Parameter Distribution: Parkinson's Disease. A) Model parameters for the best-fit model (Model 4). Bootstrapped (10,000 samples) marginal probability distributions of each parameter value are shown along the diagonal (red). 95% confidence intervals are indicated with dark grey lines. Thick dark red lines correspond to the cumulative distribution. We used the median value for $\sigma^{M,x}$ (0.41 cm), $\sigma^{M,y}$ (0.63 cm), $\sigma^{E,x}$ (0.38 cm), $\sigma^{E,y}$ (1.51 cm), α^x (0.99), α^y (0.52), $\beta^{aim,x}$ (0.23), $\beta^{target,x}$ (0.16), $\beta^{aim,y}$ (0.25) to run simulations of the Parkinson's group in **Experiment 3** with the best-fit model. Off-diagonal plots show joint probability distributions for each pair of parameters. As a reminder, the Parkinson's disease group and age-matched control group share the same movement variability terms ($\sigma^{M,x}$, $\sigma^{M,y}$, $\sigma^{E,x}$, $\sigma^{E,y}$). Notably, the median value for reinforcement-based movement updates along the task-redundant dimension in the Parkinson's disease group (α^y: 0.62) is lower than the median value for the age-matched control group (α^y: 0.90), supporting the idea that Parkinson's disease reduces the knowledge of exploratory movement variability used to update reach aim.

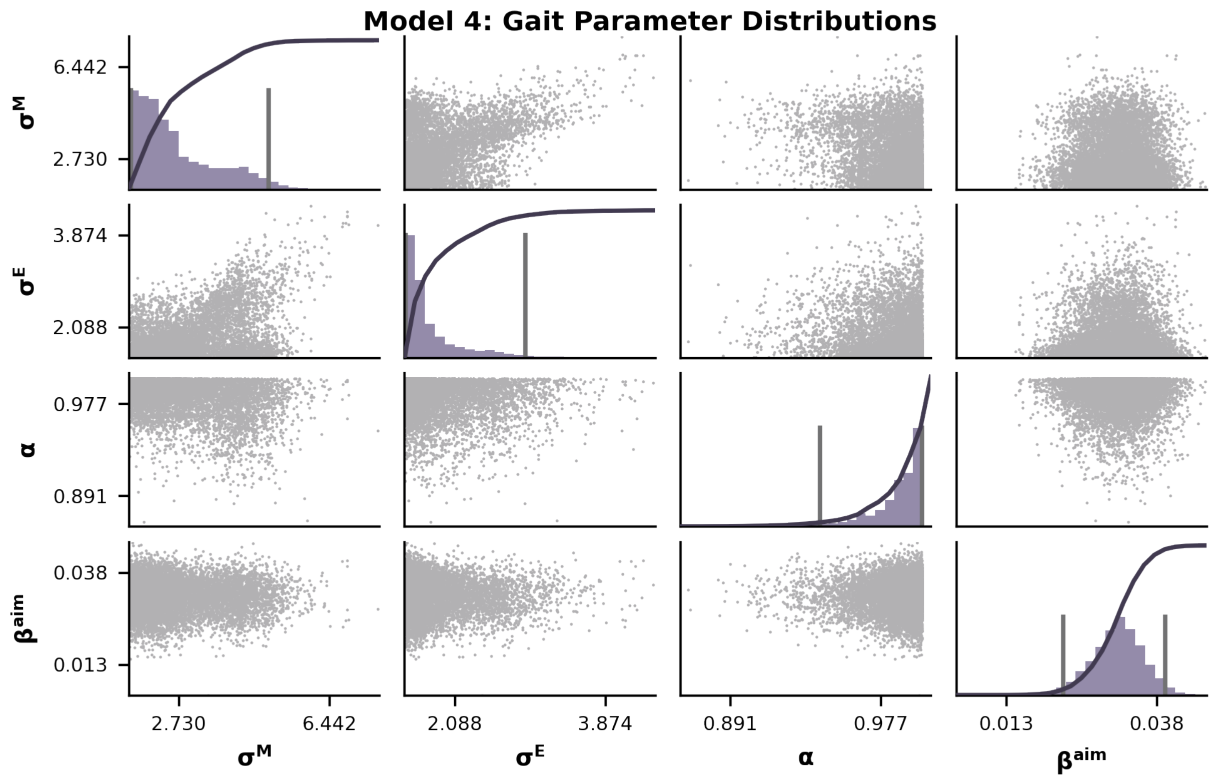

Fig L: Best-fit Model Parameter Distribution: Wood et al., 2024. Model parameters for the best-fit model (Model 4). Bootstrapped (10,000 samples) marginal probability distributions of each parameter value are shown along the diagonal (purple). 95% confidence intervals are indicated with dark grey lines. Thick dark purple lines correspond to the cumulative distribution. We used the median value for σ^M (2.3 m), σ^E (1.7 m), α (0.99), β^{aim} (0.03) to run simulations of Wood and colleagues (2024). Off-diagonal plots show joint probability distributions for each pair of parameters.

Supplementary F: Similarity between van Beers et al. (2013) Model and Correction to Intended Aim

As a part of our modelling analysis, we investigated the error signals utilized by the sensorimotor system along task-redundant dimensions. Our model selection analysis chose Model 4 to be our best-fit model for the data. Along the task-redundant dimension (Y), the best-fit model (Model 4) uses the difference between the executed movement and the previously intended movement as an error correction signal. Although conceptually different, this result is mathematically similar to the model proposed by van Beers and colleagues (2013). The van Beers model can be described in our notation as

$$Y_t = Y_t^{aim} + \epsilon_t^M + \epsilon_t^P \tag{1}$$

$$Y_{t+1}^{aim} = Y_t^{aim} + \epsilon_t^P \tag{2}$$

In the van Beers (2013) model, the executed movement along the task-redundant dimension (Y_t) is equal to the intended movement (Y_t^{aim}) plus additional motor movement variability (ϵ_t^M) and planned movement variability (ϵ_t^P). The van Beers (2013) model does not make error corrections along the task-redundant dimension. Instead, planned movement variability accumulates over trials to influence the next intended movement (Y_{t+1}^{aim}). In the van Beers model (2013), this accumulation of planned movement variability results in an exploratory random walk.

Building off our prior work [1], our best-fit model (Model 4) does not consider planned movement variability (ϵ_t^P). Additionally, our best-fit model (Model 4) uses the difference between the executed movement and the previously intended movement as an error correction signal. This formulation of error is similar to a sensory prediction error [13]. Despite utilizing an error signal, our best-fit model uses a portion of the movement variability used to execute the movement (Y_t) to update the intended movement (Y_{t+1}^{aim}), similar to the van Beers (2013) model. Below we describe the task-redundant dimension (Y) of our best-fit model (Model 4) when no reinforcement feedback is provided:

$$Y_t = Y_t^{aim} + \epsilon_t^M + (1 - r_{t-1})\epsilon_t^E \tag{3}$$

$$Y_{t+1}^{aim} = Y_t^{aim} - \beta^{aim,Y}(Y_t - Y_t^{aim}) \tag{4}$$

Observe that (3) can be re-written as

$$(Y_t - Y_t^{aim}) = \epsilon_t^M + (1 - r_{t-1})\epsilon_t^E \tag{5}$$

Substituting (5) into (4) yields

$$Y_{t+1}^{aim} = Y_t^{aim} - \beta^{aim,Y}(\epsilon_t^M + (1 - r_{t-1})\epsilon_t^E) \tag{6}$$

Note that (2) and (6) both update intended movement aim (Y_t^{aim}) by adding a portion of the movement variability that produced the executed movement (Y_t). Unlike the van Beers (2013) model which accumulates planned movement variability, our model formulation accumulates both motor and exploratory movement variability to produce exploratory random walk behaviour. As mentioned in our prior work [1], it is unclear whether planned movement variability arising from the premotor cortex [14, 15] and

exploratory movement variability arising from the basal ganglia [16, 17, 18] are unrelated processes. Indeed, the premotor cortex and basal ganglia are known to be linked through a neural loop [19, 20]. Thus, it would be useful for future work to further investigate how motor, planned, and exploratory movement variability individually contribute to sensorimotor behaviour.

Supplementary G: Absolute Change in Reach Position

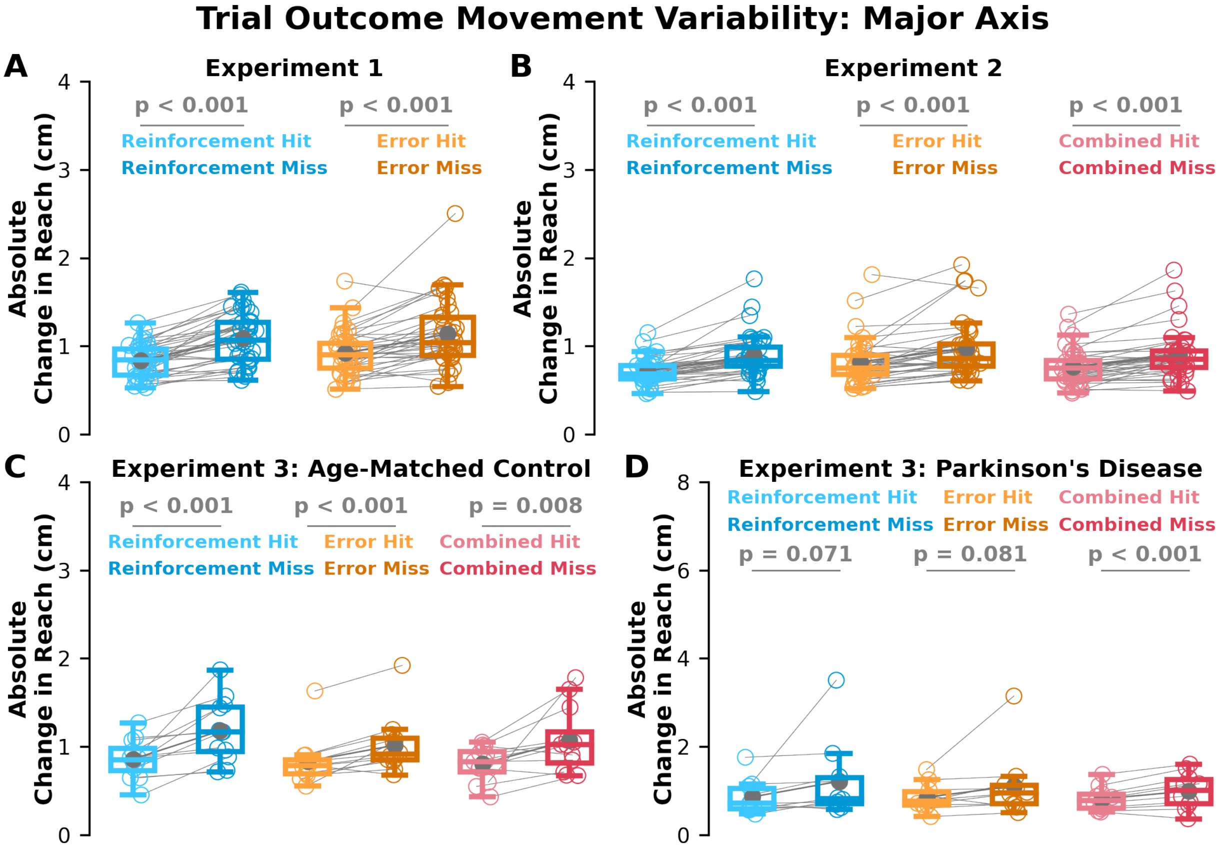

Fig M: Absolute Change in Reach Position When Comparing Hits and Misses. We calculated the absolute change in reach position from trail t and t+1 (y-axis) for each condition (x-axis) when trial t was a hit (lighter colours) or miss (darker colours). As expected, we found that neurotypical young adults in **A) Experiment 1** and **B) Experiment 2**, as well as age-matched controls in **C) Experiment 2**, all had greater absolute changes in reach position following a miss compared to a hit. **D)** For participants with Parkinson's disease in **Experiment 3**, we also found greater absolute changes in reach position following a miss in the combined reinforcement and error condition. These results suggest that participants display greater changes in reach aim in the task-redundant dimension when there is a miss along the task-relevant dimension.

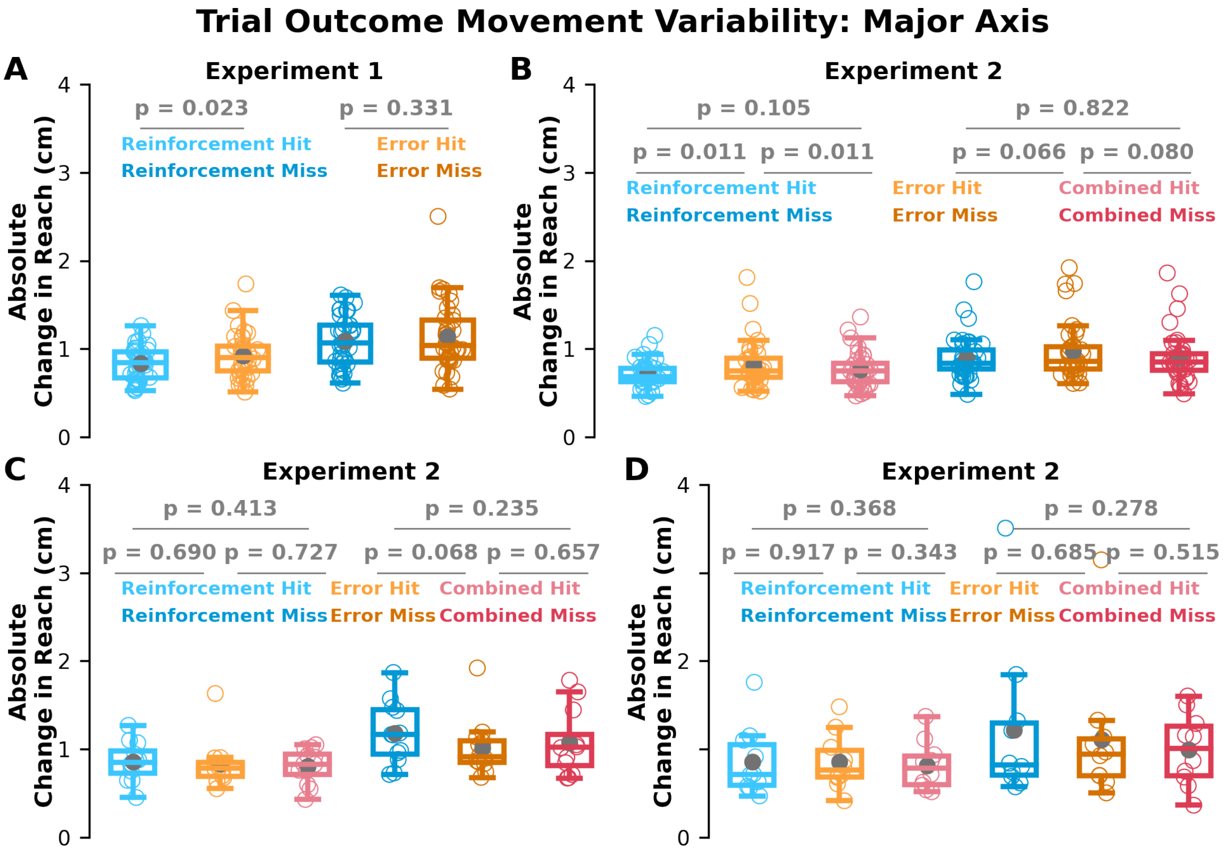

Fig N: Absolute Change in Reach Aim When Comparing Between Conditions. We calculated the absolute change in reach position from trail t and t+1 (y-axis) for each condition (x-axis) when trial t was a hit (lighter colours) or miss (darker colours). For neurotypical young adults in **A) Experiment 1** and **B) Experiment 2**, following a hit we found that there was a smaller absolute change in reach position in conditions with reinforcement feedback (reinforcement, combined) compared to the error condition. We did not see any differences between conditions for the **C)** age-matched controls or **D)** Parkinson's disease participants, which may be due to a lack of statistical power or the influence of age. For young neurotypical participants, smaller changes following a hit in conditions involving reinforcement feedback can be readily interpreted as repeating movement that have just been rewarded.

REFERENCES

1. Roth, A. M., Calalo, J. A., Lokesh, R., Sullivan, S. R., Grill, S., Jeka, J. J., Kooij, K. van der, Carter, M. J., & Cashaback, J. G. A. (2023). Reinforcement-based processes actively regulate motor exploration along redundant solution manifolds. *Proceedings of the Royal Society B: Biological Sciences*, 290 (2009), 20231475.
2. Roth, A. M., Lokesh, R., Tang, J., Buggeln, J. H., Smith, C., Calalo, J. A., Sullivan, S. R., Ngo, T., Germain, L. S., Carter, M. J., & Cashaback, J. G. A. (2024). Punishment Leads to Greater Sensorimotor Learning But Less Movement Variability Compared to Reward. *Neuroscience*, 540 , 12–26.
3. Beers, R. van, Brenner, E., & Smeets, J. (2013). Random walk of motor planning in task-irrelevant dimensions. *Journal of neurophysiology*, 109 (4), 969–977.
4. Cashaback, J., Lao, C., Palidis, D., Coltman, S., McGregor, H., & Gribble, P. (2019). The gradient of the reinforcement landscape influences sensorimotor learning. *PLoS Computational Biology*, 15 (3), 1006839.
5. Cardis, M., Casadio, M., & Ranganathan, R. (2018). High variability impairs motor learning regardless of whether it affects task performance. *Journal of Neurophysiology*, 119 (1), 39–48.
6. Dingwell, J., John, J., & Cusumano, J. (2010). *Do humans optimally exploit redundancy to control step variability in walking? PLoS computational biology*.
7. Hausdorff, J. M., Peng, C. K., Ladin, Z., Wei, J. Y., & Goldberger, A. L. (1995). Is walking a random walk? Evidence for long-range correlations in stride interval of human gait. *Journal of Applied Physiology (Bethesda, Md.: 1985)*, 78 (1), 349–358.
8. Hausdorff, J. M. (2007). Gait dynamics, fractals and falls: finding meaning in the stride-to-stride fluctuations of human walking. *Human movement science*, 26 (4), 555–589.
9. Therrien, A., Wolpert, D., & Bastian, A. (2016). Effective reinforcement learning following cerebellar damage requires a balance between exploration and motor noise. *Brain*, 139 (1), 101–114.
10. Pekny, S., Izawa, J., & Shadmehr, R. (2015). Reward-dependent modulation of movement variability. *Journal of Neuroscience*, 35 (9), 4015–4024.
11. Bakkum, A., Donelan, J. M., & Marigold, D. S. (2020). Challenging balance during sensorimotor adaptation increases generalization. *Journal of Neurophysiology*, 123 (4), 1342–1354.
12. — (2021). Savings in sensorimotor learning during balance-challenged walking but not reaching. *Journal of Neurophysiology*, 125 (6), 2384–2396.
13. Shadmehr, R., Smith, M. A., & Krakauer, J. W. (2010). Error Correction, Sensory Prediction, and Adaptation in Motor Control. *Annual Review of Neuroscience*, 33 (1), 89–108.
14. Churchland, M., Afshar, A., & Shenoy, K. (2006). A central source of movement variability. *Neuron*, 52 (6), 1085–1096.
15. Sutter, K., Oostwoud Wijdenes, L., Beers, R. J. van, & Medendorp, W. P. (2021). Movement preparation time determines movement variability. *Journal of Neurophysiology*, 125 (6), 2375–2383.

16. Fee, M., & Goldberg, J. (2011). A hypothesis for basal ganglia-dependent reinforcement learning in the songbird. *Neuroscience*, 198 , 152–170.
17. Olveczky, B., Andalman, A., & Fee, M. (2005). Vocal experimentation in the juvenile songbird requires a basal ganglia circuit. *PLoS biology*, 3 (5), 153.
18. Kao, M., & Brainard, M. (2006). Lesions of an avian basal ganglia circuit prevent context-dependent changes to song variability. *Journal of neurophysiology*, 96 (3), 1441–1455.
19. Alexander, G. E., DeLong, M. R., & Strick, P. L. (1986). Parallel Organization of Functionally Segregated Circuits Linking Basal Ganglia and Cortex. *Annual Review of Neuroscience*, 9 (1), 357–381.
20. Middleton, F. A., & Strick, P. L. (2000). Basal ganglia and cerebellar loops: motor and cognitive circuits. *Brain Research Reviews*, 31 (2), 236–250.
